# Conserved 2nd Residue of Helix 8 of GPCR May Confer the Subclass-Characteristic and Distinct Roles through a Rapid Initial Interaction with Specific G Proteins

**DOI:** 10.3390/ijms20071752

**Published:** 2019-04-09

**Authors:** Takaaki Sato

**Affiliations:** Biomedical Research Institute, National Institute of Advanced Industrial Science and Technology, 1-8-31 Midorioka, Ikeda, Osaka 563-8577, Japan; taka-sato@aist.go.jp; Tel.: +81-72-751-8342

**Keywords:** G protein-coupled receptor, G protein subtypes, human, classification, initial specific interaction, helix 8, hydrophobic core

## Abstract

To obtain a systematic view of the helix-8-second residue responsible for G protein-coupled receptor (GPCR)–G protein initial specific interactions, 786 human GPCRs were subclassified based on the pairs of agonist groups and target G proteins and compared with their conserved second residue of helix 8. Of 314 non-olfactory and deorphanized GPCRs, 273 (87%) conserved single amino acids in the subclasses, while 93 (58%) of the 160 subclasses possessed only a single GPCR member. Class B, C, Frizzled, and trace amine-associated GPCRs demonstrated 100% conservation, whereas class I and II olfactory and vomeronasal 1 receptors demonstrated much lower rates of conservation (20–47%). These conserved residues are characteristic of GPCR classes and G protein subtypes and confer their functionally-distinct roles.

## 1. Introduction

In humans, nearly 800 G protein-coupled receptors (GPCRs) detect various extracellular physiological or environmental signal molecules. These range widely from atomic ions to structural features of proteins. Activated GPCRs activate one or a few of the 16 G proteins for one or more distinct cellular responses, leading to regulations of various internal physiological systems such as cardiovascular, neural, immune, sensory, hormonal, and differentiation systems [1]. GPCRs are typically classified into eight classes: class A (279 non-olfactory members, 52 class I and 333 class II olfactory receptors (ORs), 6 trace amine-associated receptors (TAARs)), class B (15 members), class C (23 members), adhesion class (33 members), Frizzled class (11 members), vomeronasal type 1 (VN1, 5 members), Taste2 (TAS2, 25 members), and the other GPCRs (4 members) [1,2]. Evolutionarily, the divergence of class A GPCRs is expanded in multicellular animals, whereas unicellular organisms mainly possess class B and class C GPCRs [3]. G proteins are grouped into four classes: G_s_ class (G_s_, G_olf_), G_i/o_ class (G_i1_, G_i2_, G_i3_, G_t1_, G_t2_, G_t3_, G_o_, G_z_), G_q/11_ class (G_q_, G_11_, G_14_, G_15_), and G_12/13_ class (G_12_, G_13_) [3], and contrastingly, unicellular organisms have representatives of all four human G protein classes [3]. The crystal structures of the active-state rhodopsin and β_2_ adrenergic receptor (β_2_AdR) led to the discovery of the common rearrangement mechanism behind the intramolecular interaction of GPCR during its activation [4,5,6]. However, the molecular mechanism underlying specific interactions between GPCRs and G protein remains unclear, except for the selectivity barcode of 25 amino acids in G proteins [3].

**Figure 1:**
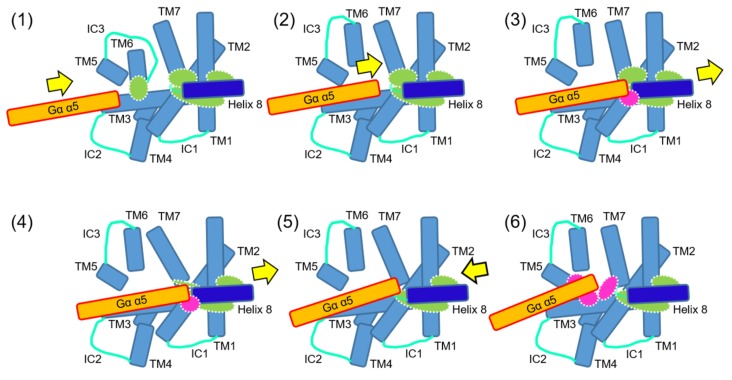
A transition model of multistep interactions between GPCR and G protein [10]. The cytoplasmic view of a possible sequential interaction process is shown. Intramolecular interactions (green closed circles) and intermolecular interactions (magenta closed circles) are broken, or maintained, or formed.

A systematic analysis of a chimeric G protein and scanning mutagenesis of a GPCR has shed light on responsible residues for the specific interaction. The replacement of the non-olfactory Gα_15_ C-terminal six amino acids, ^369^DEINLL, with the corresponding Gα_olf_, ^376^KQYELL, improved the interaction specificity between OR-S6 and Gα_15_ [7]. This chimeric G protein mediated a more rapid (2.2-fold) and robust (1.7-fold) Ca^2+^ response in a HEK293 functional expression system [7,8]. Regarding responsible residues of the GPCR, the second residue of helix 8 of OR-S6 was identified, by observing the complete loss of improved response dynamics in an alanine-scanning and charge-altering mutagenesis of OR-S6 helix 8 with the chimeric Gα_15-olf_. The homology modeling indicates that the specific interaction between OR-S6 and Gα_15-olf_ is based on the stabilized intracellularly-superficial configuration of the helix-8-second residue by the hydrophobic core between helix 8 and transmembrane domain 1–2 (TM1–2) [8]. Then, the initial, transient, and specific interaction between a GPCR, OR-S6, helix-8-second residue (Glu) and Gα_15-olf_ C-terminal sixth residue (Lys) was predicted and supported by a high conservation of helix-8-second residues in the GPCR subclasses based on pairs of agonist groups and G protein subtypes for 178 non-olfactory GPCRs [9]. Moreover, its functional importance is supported by an almost identical class-dependent occupancy of helix-8-second-Glu class I and II ORs in humans and mice [9]. Moreover, a transition step model from an inactive state to a stable interaction via an initial, transient, and specific inter-helical interaction was proposed (Figure 1) [10]. The model starts with the inactive-state crystal structure of β_2_AdR and ends with its active-state crystal structure of the complex with the extended interactions between GPCR and G protein through possible intermediate processes, which facilitate breaking some of the inactive-state intra-molecular interactions.

In the present study, to obtain a systematic view of the helix-8-second residue responsible for the GPCR–G protein initial specific interactions, our previous analysis of conserved helix-8-second residues was expanded to 786 human GPCRs. The conserved helix-8-second residues are characteristic of GPCR classes and specific G protein subtypes. These results suggest that the conserved helix-8-second residues confer functionally distinct roles in parallel GPCR signaling. The initial and subclass-characteristic transient process can be a potential drug target for specific GPCR-regulated signaling pathways.

## 2. Results and Discussion

### 2.1. High Conservation of Helix-8-Second Residues of GPCRs in Subclasses Except for ORs and VN1 Receptors

In the Supplementary Materials, the sequences of TM7 NPxxY or corresponding motifs and adjacent helix 8 of GPCRs and their target G proteins are shown for non-olfactory and deorphanized class A GPCRs (204 members: 194 and 10 GPCRs with identified and not identified G proteins, respectively, Figure S1), class B GPCRs (15 members, Figure S2), deorphanized class C GPCRs (15 members, Figure S3), adhesion class GPCRs (33 members, Figure S4), Frizzled class GPCRs (11 members, Figure S5), VN1 receptors (5 members) and other GPCRs (4 members, Figure S6), TAS2 GPCRs (25 members, Figure S7), non-olfactory and orphan class A GPCRs (75 members, Figure S8), and orphan class C GPCRs (8 members, Figure S9). They were subclassified based on GPCR classes and pairs of agonist group and G protein subtypes (Tables S1–S5).

The rates of GPCRs with a conserved helix-8-second residue in each subclass are summarized in Table 1, except for 75 members of non-olfactory and orphan class A GPCRs and eight members of class C GPCRs. Of the 314 non-OR and deorphanized GPCRs (160 subclasses), 273 (87%) (135 subclasses; 84%) conserved single helix-8-second residues in the subclasses. Class B, C, and Frizzled GPCRs and TAARs demonstrated 100% conservation. The high conservation of GPCR helix-8-second residues suggests their important subclass-dependent role in GPCR signaling.

Why does the GPCR signaling system require the conserved helix-8-second residues? As described previously [9,10], both GPCRs and G proteins are activated in two-step processes, which are an initial, transient, and specific interaction and a subsequent GPCR-common and stable interaction. The initial interaction is likely an activation rapidity determinant, whereas the stable one is likely formed in an initial interaction-dependent manner. The main determinant of GPCR signaling is likely agonist affinities of GPCRs. In addition, when agonist–GPCR binding affinities are similarly high, GPCR–G protein interaction specificities must be critical for rapid and robust cellular responses. Although only one scanning mutagenesis analysis has concluded that the helix-8-second residue is a determinant for the initial, transient, and specific interaction of a GPCR and a chimeric G protein, the high conservation of the helix-8-second residue in the deorphanized GPCR subclasses strongly supports that a single residue at the second position of helix 8 governs cellular response rapidity via GPCR–G protein initial interaction specificities. In other words, the conserved helix-8-second residue could simply assure an agonist binding affinity-dependent cellular response in parallel GPCR signaling pathways via a uniform activation rapidity of a target G protein.

### 2.2. GPCR Class- and G Protein Subtype-Characteristics of Conserved Helix-8-Second Residues

In contrast, class I and II ORs and VN1Rs demonstrated much lower conservation rates (20–47%) of helix-8-second residues (Table 1). These differences are attributable to a genetic origin and species dependency. In humans, helix-8-second-Glu ORs are 23% and 47% in classes-I and class-II ORs, respectively, whereas helix-8-second Asp ORs are 0% and 42%, respectively, with high cross-species conservation between human and mouse [9,10]. However, pheromone receptors, human VN1Rs and murine *m*Vmn1rs, form a more species-specific family (Table 2), consistent with the previous report [11].

In mice, 43% of 112 *m*Vmn1rs, which interact with G_i2_, conserved Arg and no helix-8-second-Glu, Gln, or Asp *m*Vmn1rs, whereas human VN1Rs equally conserved Arg (20%), His (20%), and Gln (20%) (Table 2, Figure S6). Notably, 117 *m*Vmn1rs, which showed no characteristic features of helix 8, were excluded. Considering the lack of helix-8-second-Arg ORs and the predicted initial, transient, and specific, inter-helical, and ionic interaction between GPCR helix-8-second-Glu and G_olf_ C-terminal sixth Arg, the positively-charged helix-8-second-Arg would specifically interact with G_i2_ C-terminal fifth Asp (Figure S10). Similarly, four subclasses of chemokine receptors, which conserve Lys (60–100%), would also specifically interact with G_i_ C-terminal fifth Asp or Glu (Table S1, Figures S1 and S10). Moreover, conserved Lys in bitter tastant TAS2 receptors (76%) indicates an initial and specific interaction between GPCR helix-8-second Lys and G_t3_ C-terminal fifth Asp (Table 2, Figure S10).

Thus, the present analysis complemented the results from a previous study [10], where highly-conserved helix-8-second residues are characteristic of GPCR subclasses, i.e., characteristic of GPCR classes and G protein subtypes (Table 2). Markedly, Trp is conserved at the second position of helix 8 only in TAARs, which mediate aversive responses to odors, in both humans and mice [10], but not in all the other GPCRs. Non-olfactory class-A GPCRs similarly conserved helix-8-second Glu (16%), Asp (13%), Asn (15%), and Lys (14%). This contrasts with the uneven rates of helix-8-second Glu, Gln, and Asp in class I and II ORs (Table 2). Moreover, the highly-conserved helix-8-second residues were Glu (100%) for class B GPCRs, Asn (60%) for class C GPCRs, Glu (24%) and Lys (27%) for adhesion class GPCRs, and Lys (91%) for Frizzled class GPCRs, whereas no characteristic residues were observed for orphan class A and C GPCRs and G-protein-unknown, non-olfactory class A GPCRs (Table 2, Figures S8 and S9). In non-OR GPCRs, the most conserved helix-8-second residue was Lys (for 75 GPCRs = 82 − 1 − 6), consistent with a high rate of 11/16 G proteins for negatively-charged Asp or Glu at the C-terminal fifth or sixth position of α5 compared to 5/16 for non-charged residues.

To shed light on the initial interaction specificities between GPCRs and G proteins, the differences in conserved helix-8-second residues between G protein subtypes were further analyzed in non-olfactory and deorphanized class A, class B, and class C GPCRs. The GPCRs with each helix-8-second residue were summed for combinations of target G protein subtypes in each class (Table S6). In G protein subtypes, the conserved helix-8-second residues were GPCR class-dependent and characteristic of G_s_ (Glu (33%) and Asp (38%) in class A vs. Glu (100%) in class B), G_i/o_ (Lys (25%) and Asn (23%) in class A vs. Asn (100%) class C), and G_q/11_ (Glu (19%), Arg (14%), His (14%), and Ser (14%) in class A vs. Glu (40%) and Asn (40%) in class C). 

Common to class A and B GPCRs likely coupling to G_s_, a highly-conserved helix-8-second residue was the negatively-charged Glu, supporting the initial and specific interaction with the positively-charged Arg at the sixth position of the C-terminal. Similarly, Glu was highly and commonly conserved for class A and C GPCRs, which interact only with G_q/11_, suggesting an initial, transient, and specific interaction with G protein C-terminal sixth Lys. However, class A and C GPCRs interacting only with G_i/o_ commonly conserved non-charged polar helix-8-second-Asn, a specific interaction partner of which was not predicted. In addition to Asn, class A GPCRs for G_i/o_ similarly conserved positively-charged Lys, which was able to form initially a specific interaction with the C-terminal fifth negatively-charged Asp or Glu in a manner similar to that of TAS2 GPCRs.

Notably, based on the stable interaction between M3R helix-8-first Lys and G_q_ loop G.h4s6.12 Asp (common Gα numbering system) [12,13], a non-specific or less specific, stable, and loop-helical interaction between GPCR helix-8-first Lys and G protein G.h4s6.10 or G.h4s6.12 Asp was predicted [10]. This is supported by the charge-altering mutant OR-S6-impaired response rapidity [8,9,10]. In a future study, a scanning mutagenesis analysis would validate this model by running a comparison between the relative contributions of G protein C-terminal sixth and fifth residues to the initial interactions with these GPCR helix-8-second residues.

### 2.3. Key GPCRs for the Determination of Detected Physiological and/or Biological Information in Parallel GPCR Signaling

The principles are generally simple, but hidden by complicated phenomena under additional heterogeneous conditions. A single residue-determined GPCR–G protein interaction specificity is a potential candidate for the principle in parallel GPCR signaling due to its simplicity. Highly-conserved helix-8-second Glu was overlapped between GPCRs for G_s_ and G_q/11_. However, the cell type-specific expression of the G protein subtype could prevent the overlapping of helix-8-second Glu from mediating cross-talk between parallel GPCR–G_s_ and G_q/11_ signaling pathways. If this is the case, the GPCR subclass-characteristic, i.e., specific G protein-characteristic helix-8-second residues, would strengthen the transition step model of the GPCR–G protein initial, transient, specific, and inter-helical interaction into a common, stable, extended interaction for a single-residue-determined uniform activation rapidity of a target G protein [9,10,14]. The helix-8-second residue could determine within-subclass-distinct functional roles of GPCRs in parallel GPCR signaling.

Next, we determined which GPCR with a conserved helix-8-second residue is key to controlling parallel GPCR-mediated regulations of multiple physiological systems. The GPCRs, which are most sensitive to a given agonist and most specific for a target G protein, most rapidly activate the G protein and subsequently induce the most robust cellular responses. Such a rapid and robust response could be a determinant in the parallel GPCR-mediated regulations or signaling. GPCRs that mediate such determinant responses are defined as key GPCRs. Among the GPCR subclasses, the most difficult question is “Which residue do key ORs conserve at the second position of helix 8?” Considering the specific conditions and evidence for the OR subfamily and the olfactory neural system, the principle would address this question.

The present analysis confirmed that the dual multiple subclasses of the OR subfamily are unique in GPCR subfamilies [9,10]. GPCRs with a rapidly-interacting, subclass-dependent, and highly-conserved helix-8-second residue are likely to be key GPCRs. Glu is the only rapidly-interacting and highly-conserved helix-8-second residue common between class I and II ORs. A series of point mutations at the helix-8-second residue in OR-S6 indicates the importance of the negative charge for cellular response rapidity via the chimeric Gα_15-olf_. Although Asp is also negatively charged, there are no class I helix-8-second-Asp ORs [9,10]. Furthermore, our transition step model of the GPCR–G interaction [10] predicts the advantage of Glu for a rapid activation. In the homology modeling of OR-S6 and the transition step model [9,10], the negatively-charged atom of Glu at the second position of helix 8 is one carbon chain-length closer to the accessing C-terminal region of G protein α5, suggesting a more rapid initiation of the initial interaction between GPCR helix 8 and G protein α5. These differences and the following architecture of the odor information processing both suggest that helix-8-second-Glu ORs are key ORs rather than auxiliary ORs. The architecture of the odor information processing demonstrates why response rapidity is very important in the olfactory system [9,10,14].

A signal of an odorant is detected in the olfactory sensory neuron (first neuron), which transfers the OR signal to mitral and tufted cells (second neurons) in the first olfactory center via one or two OR-specific relay points. The third neurons of the olfactory pathway are distributed in the second olfactory centers. As one of them, pyramidal cells in the anterior piriform cortex integrate signals from multiple cognate ORs by input synchrony through feedforward inhibition via the more sensitive tufted-cell pathway [15,16]. These integrated signals are characteristic of distinct odors, likely representing elemental odors (corresponding to the R/G and Y/B elemental colors primarily extracted in the visual third neurons under inhibitory conditions) [9,10,14]. Notably, wavelet correlation analysis revealed that input and output signals of the third neurons change in information redundancy [17].

A change in initially-activated key ORs could alter perceived odors in a hierarchical elemental-odor coding manner. The odor mixture-dependent stress relaxation indicates a hierarchy of elemental odors: rose odor > fox-unique 2,4,5-trimethyl thiazoline (TMT) odor > caraway odor [18,19]. The less sensitive key OR for TMT likely provides an explanation for the above and associated results. By the genetic ablation of all dorsal ORs, ΔD mice are unable to recognize fox-unique TMT odor, although they retain the high sensitivity to TMT [20]. The most sensitive OR for TMT odor is helix-8-second-Asp OR [21], while only one helix-8-second-Glu OR, as a candidate of key OR for TMT, is less sensitive than the other three helix-8-second-Asp ORs [10]. The deletion of the less-sensitive key OR results in the impaired recognition of TMT odor and the maintained high sensitivity to TMT via the remaining highly-sensitive helix-8-second-Asp ORs [10]. Early inputs from key ORs to the third neurons in the ventrorostral region of the anterior piriform cortex would coordinate the integration of cognate OR signals for rose odor earlier than those of TMT odor through input synchrony by the feedforward inhibitory signals delivered entirely within the anterior piriform cortex, resulting in the higher ranked hierarchy of rose odor compared to TMT odor [10,14].

In contrast, all human and murine ORs that are most sensitive to an elementally-resistant odor of musk, which is used as base notes in many perfumes, are helix-8-second-Glu ORs [22]. Moreover, the deletion of the most sensitive helix-8-second-Glu OR for R-(−)-carvone and the maintenance of the most sensitive helix-8-second-Glu and R-(−)-/S-(+)-carvone-non-discriminating OR could explain an inability to distinguish between R-(−)- and S-(+)-carvone with a retained high sensitivity to R-(−)-carvone [14]. These results strongly suggest that helix-8-second-Glu ORs are key ORs for the determination of odor representation. Helix-8-second-Gln ORs and helix-8-second-Asp ORs could therefore contribute to odor decoding as auxiliary elemental odors and odor detection sensitizers, respectively, in the brain [9,10].

Thus, conserved helix-8-second residues confer functionally-distinct roles in parallel GPCR signaling. In both ORs and non-OR GPCRs, such as the three adrenergic receptor subclasses, the signal/elemental information hierarchy will be validated in future studies. Analysis of response kinetics using chimeric Gα_15_ proteins by replacing the C-terminal six residues of each G protein subtype would be useful to validate the transition step model for rapid and specific activations of GPCRs and G proteins. Moreover, analysis of residues for the extended stable interaction between GPCRs and G proteins using scanning mutagenesis would be required to understand fully the molecular mechanism underlying GPCR–G protein interaction specificities and hierarchical GPCR signal processing.

## 3. Materials and Methods

### 3.1. Subclassification of GPCRs

Sequences of TM7 and helix 8 for target G protein subtypes were analyzed for non-olfactory GPCRs (204 deorphanized + 75 orphan members), class B GPCRs (15), class C GPCRs (15 deorphanized + 8 orphan), adhesion class GPCRs (33), Frizzled class GPCRs (11), and other GPCRs (4) in humans using data from the IUPHAR/BPS Guide to Pharmacology database [1] and its linked webpage at the Universal Protein Resource (https://www.uniprot.org/) or data from our previous papers with some corrections [9,10]. The sequences of an OR (OR2AT4), VN1 receptors (5 VN1Rs and 229 murine mVmn1rs, including 18 predicted murine members), and TAS2 receptors (24) in humans were obtained from the NCBI gene database [2] and its linked webpage at the Universal Protein Resource (https://www.uniprot.org/). The other GPCRs (52 class I and 332 class II ORs, 6 TAARs) were re-used from our previous papers [9,10]. All GPCRs were subclassified based on their classes, agonist groups, and target G proteins.

### 3.2. Alignment of TM7 NPxxY Motif and Helix 8 of GPCR

The alignment of the TM7 NPxxY motif and helix 8 of GPCRs was manually achieved based on the sequence features observed in our reported homology modeling of OR-S6 [8]. Helix 8 was predicted based on two criteria: (i) hydrophobic residues at the 3rd, 7th, and/or 8th and 10th and/or 11th position of helix 8 in the C-terminal region of the GPCR and (ii) the 2nd residue of helix 8 located at the 7th or 8th position from the last Tyr residue of the NPxxY motif or those of other corresponding and conserved motifs located close to the C-terminus of TM7. Hydrophobic residues for the hydrophobic core between helix 8 and TM1–2 were predicted based on the positions of those in the OR-S6 [8]. Some of the predicted helix 8 and their instability in previously-reported GPCRs [10] were modified in the present study.

## Figures and Tables

**Table 1:** Rate of human GPCRs with conserved helix-8-second residue in agonist–G protein pair-based subclasses.

GPCR Class	Class A				Class B GPCRs	Class C GPCRs	Adhesion Class GPCRs	Frizzled Class GPCRs	VN1 GPCRs	TAS2 GPCRs	Non-OR GPCRs
	Non-Olfactory Class A GPCRs	Class-I ORs	Class-II ORs	TAARs							
All GPCRs ^†^ (subclasses)	204 (117)	52 (1)	333 (1)	6 (1)	15 (10)	15 (7)	33 (16)	11 (7)	5 (1)	25 (1)	314 (160)
Conserved GPCRs ^‡^ (subclasses)	179 (98)	12 (0)	156 (0)	6 (1)	15 (10)	14 (6)	28 (13)	11 (7)	1 (0)	19 (0)	273 (135)
Conserved GPCR rate (subclass rate)	88% (84%)	23% (0%)	47% (0%)	100% (100%)	100% (100%)	93% (86%)	85% (81%)	100% (100%)	20% (0%)	76% (0%)	87% (84%)

GPCR, G protein-coupled receptor; OR, olfactory receptor; TAAR, trace amine-associated receptor; VN1, vomeronasal type 1; TAS2, Taste2. Human GPCRs were subclassified by pairs of agonist group and G-protein subtypes. Orphan class A and class C GPCRs ^†^ were excluded. Helix-8-2nd-Glu class I and II ORs were counted for single-amino acid-conserved GPCR ^‡^.

**Table 2:** Classification of olfactory receptors and other GPCRs by helix-8-second residues and subtypes of G proteins (modified from [9,10]).

GPCRs and Their Rates		Helix-8 Second Residue									2nd Residue of Helix 8 (GPCR Number)	NPxxY Motif
	All	Glu	Gln	Asp	Asn	His	Lys	Arg	Trp	Others		
non-olfactory class A GPCRs’	194	**31**	5	**26**	**30**	7	**28**	19	**0**	48	S(15), T(12), G(6), A(5), P(4), I(2), L(1), V(1), F(1), no h8(1)	(N/D)PxxY, (N/D)PxxF
rate (misc)	100%	**16%**	3%	**13%**	**15%**	4%	**14%**	10%	**0%**	25%		
class-I ORs’	52	**12**	**36**	0	0	0	1	0	**0**	2	T(1), P(1)	NPxxY
rate (G_olf_)	100%	**23%**	**69%**	0%	0%	0%	2%	0%	**0%**	4%		
class-II ORs’	333	**156**	22	**139**	1	2	6	0	**0**	7	A(3), V(1), T(1), S(1), M(1)	
rate (G_olf_)	100%	**47%**	7%	**42%**	0%	1%	2%	0%	**0%**	2%		
TAARs’	6	0	0	0	0	0	0	0	**6**	0		
rate	100%	0%	0%	0%	0%	0%	0%	0%	**100%**	0%		
class B GPCRs’	15	**15**	0	0	0	0	0	0	**0**	0		V(A/S)xxY
rate (G_s_, G_s_ > G_q/11_, ‒)	100%	**100%**	0%	0%	0%	0%	0%	0%	**0%**	0%		
class C GPCRs’	15	3	0	2	**9**	0	0	0	**0**	1	G(1)	PKCYxY, VYIIxF, IYIILF
rate (G_i/o_, G_q/11_, ‒)	100%	20%	0%	13%	**60%**	0%	0%	0%	**0%**	7%		
adhesion class GPCRs’	33	**8**	5	4	2	0	**9**	0	**0**	5	S(1), T(1), C(1), P(1), no helix 8(1)	Fx(V/I)xxx(H/Y)C, xFIFxF(H/Y)C, LFIFLx(H/Y)C
rate (G_q/11_, G_s_, G_12/13_)	100%	**24%**	15%	12%	6%	0%	**27%**	0%	**0%**	15%		
Frizzled class GPCRs’	11	0	0	0	0	0	**10**	0	**0**	1	A(1)	ITSxxWI, TGIAMxW
rate (G_i/o_, G_q/11_, G_s_, G_12/13_)	100%	0%	0%	0%	0%	0%	**91%**	0%	**0%**	9%		
vomeronasal 1 Rs’	5	0	1	0	0	1	0	1	**0**	2	S(2), no helix 8 in R1(H), R2(S) & R3(R)?	SPxxL
rate (G_i2_)	100%	0%	20%	0%	0%	20%	0%	20%	**0%**	40%		
murine vomeronasal 1 Rs’	112	0	0	0	0	11	15	**48**	**0**	38	L(12), T/S(7+6), I(3), F/M/Y(3+2+2), P(2)	TPLVQ, TSYSI, SPLVF, SPxVL, ITxII
rate (G_i2_)	100%	0%	0%	0%	0%	10%	13%	**43%**	**0%**	34%		
murine vomeronasal 1 Rs’	117	0	0	0	0	0	0	0	**0**	117	no helix 8	no characteristic features of helix 8
rate (G_i2_)	100%	0%	0%	0%	0%	0%	0%	0%	**0%**	100%		
Bitter tastant TAS2 Rs’	25	0	0	0	0	0	**19**	2	**0**	4	G(2), T(2)	H(S/P)xIL
rate (G_t3_)	100%	0%	0%	0%	0%	0%	**76%**	8%	**0%**	16%		
non-olfactory class A GPCRs’	10	0	1	1	1	0	1	1	**0**	5	T(2), S(1), A(1), no helix 8(1)	(N/S/T)PxxY, no NPxxY
rate (no identified G proteins)	100%	0%	10%	10%	10%	0%	10%	10%	**0%**	50%		
orphan class A GPCRs’	75	**9**	**6**	**5**	**6**	**6**	**6**	**4**	**0**	**33**	G(6), T(7), Y(1), V(2), S(8), P(4), A(2), F(1), no helix 8(2)	(N/D)PxxY, (N/D)PxxF
rate (G_q/11_, G_i/o_, G_s_, ‒)	100%	**12%**	**8%**	**7%**	**8%**	**8%**	**8%**	**5%**	**0%**	**44%**		
orphan class C GPCRs’	8	2	1	0	0	0	1	0	**0**	4	S(1),A(2), no helix 8(1)	PKCYxI, TTTxxL, no conserved motif
rate (G_i/o_)	100%	25%	13%	0%	0%	0%	13%	0%	**0%**	50%		
other GPCRs’	4	0	0	0	0	0	1	1	**0**	2	S(1), A(1)	SPxxL, TLxxF, LPxxL, SLxxY, or NCxxF
rate (?)	100%	0%	0%	0%	0%	0%	25%	25%	**0%**	50%		
all human GPCRs’	786	236	77	177	49	16	82	28	6	114		
rate (misc)	100%	30%	10%	23%	6%	2%	10%	4%	1%	15%		

## Supplementary Materials

Supplementary Materials can be found at https://www.mdpi.com/1422-0067/20/7/1752/s1.
