# Supplementary material for: Conserved 2nd Residue of Helix 8 of GPCR May Confer the Subclass-Characteristic and Distinct Roles through a Rapid Initial Interaction with Specific G Proteins
Source: Int J Mol Sci. 2019 Apr 9;20(7):1752. doi: 10.3390/ijms20071752 (PMC6480185; doi:10.3390/ijms20071752)
Supplement: Supplementary file 1 [file ijms-20-01752-s001.pdf]

Conserved Second Residue of Helix 8 of GPCR May Confer Subclass-Characteristic and Distinct Roles through a Rapid Initial Interaction with Specific G Proteins

Takaaki Sato*

Biomedical Research Institute, National Institute of Advanced Industrial Science and Technology, 1-8-31 Midorioka, Ikeda, Osaka 563-8577, Japan

*Corresponding authors. Fax: +81-6-6491-9628.

E-mail address: taka-sato@aist.go.jp

Supporting Information

Supplementary Data

Table S1–S5

Fig. S1–S10

Table S1. Classification of class A GPCRs by helix 8-second residues & subtypes of G proteins.

GPCRs	Helix-8 second residue										Predicted Hierarchy,
(signal, G protein subtypes)	all	Glu	Gln	Asp	Asn	His	Lys	Arg	Trp	misc	the 2nd residue, misc.
Rhodopsin/ Opsin1SW/MW/LW Rs	4	0	**4**	0	0	0	0	0	0	0	
(light, G_t)	100%	0%	100%	0%	0%	0%	0%	0%	0%	0%	
Opsin3/4/5 Rs	3	0	0	0	0	0	**3**	0	0	0	OPN3: ligands? G-protein subtypes?
(light?, $G_{i/o}$)	100%	0%	0%	0%	0%	0%	**100%**	0%	0%	0%	
$\beta_{1/2/3}$ Adrenergic Rs	3	0	0	**3**	0	0	0	0	0	0	
(hormone, G_s)	100%	0%	0%	**100%**	0%	0%	0%	0%	0%	0%	
$\alpha_{1A/B/D}$ Adrenergic Rs	3	**3**	0	0	0	0	0	0	0	0	
(hormone, $G_{q/11}$)	100%	**100%**	0%	0%	0%	0%	0%	0%	0%	0%	
$\alpha_{2A/B/C}$ Adrenergic Rs	3	0	0	**3**	0	0	0	0	0	0	
(hormone, $G_{i/o}$)	100%	0%	0%	**100%**	0%	0%	0%	0%	0%	0%	
Dopamine D1/5 Rs	2	0	0	**2**	0	0	0	0	0	0	
(neurotransmitter, G_s)	100%	0%	0%	**100%**	0%	0%	0%	0%	0%	0%	
Dopamine D2/3/4 Rs	3	**3**	0	0	0	0	0	0	0	0	
(neurotransmitter, $G_{i/o}$)	100%	**100%**	0%	0%	0%	0%	0%	0%	0%	0%	
Serotonin 5-$HT_{4/6/7}$ Rs	3	0	0	2	0	0	0	0	0	1	5-HT_6(D)/$_7$(D) >
(neurotransmitter, G_s)	100%	0%	0%	67%	0%	0%	0%	0%	0%	17%	5-HT_4(S) (G_s)
Serotonin 5-$HT_{1A/B/D/E/F/5A}$ Rs	6	1	0	4	1	0	0	0	0	0	
(neurotransmitter, $G_{i/o}$)	100%	17%	0%	67%	17%	0%	0%	0%	0%	0%	
Serotonin 5-$HT_{2A/B/C}$ Rs	3	0	0	0	0	0	0	0	0	3	5-HT_{2A}(T)/$_B$(T)/$_C$(I)
(neurotransmitter, $G_{q/11}$)	100%	0%	0%	0%	0%	0%	0%	0%	0%	100%	
Histamine H1 R	1	0	0	0	**1**	0	0	0	0	0	
(neurotransmitter, $G_{q/11}$)	100%	0%	0%	0%	**100%**	0%	0%	0%	0%	0%	
Histamine H2 R	1	0	0	**1**	0	0	0	0	0	0	
(neurotransmitter, $G_{q/11}$ > G_s)	100%	0%	0%	**100%**	0%	0%	0%	0%	0%	0%	
Histamine H3/4 Rs	2	0	0	0	0	0	0	1	0	1	H4(R) >
(neurotransmitter, $G_{i/o}$)	100%	0%	0%	0%	0%	0%	0%	50%	0%	50%	H3(S) (G_i)
Melanocortin MC1/2/3/4/5 Rs	5	**5**	0	0	0	0	0	0	0	0	DPxxY-motif
(hormone, G_s)	100%	**100%**	0%	0%	0%	0%	0%	0%	0%	0%	
Acetylcholine (muscarinic) MC1/3/5 Rs	3	**3**	0	0	0	0	0	0	0	0	
(neurotransmitter, $G_{q/11}$)	100%	**100%**	0%	0%	0%	0%	0%	0%	0%	0%	
Acetylcholine (muscarinic) MC2/4 Rs	2	**2**	0	0	0	0	0	0	0	0	
(neurotransmitter, $G_{i/o}$)	100%	**100%**	0%	0%	0%	0%	0%	0%	0%	0%	
Melatonin MTNR1A/B Rs	2	0	0	0	**2**	0	0	0	0	0	NAxxY motif mutant
(hormone, $G_{i/o}$)	100%	0%	0%	0%	**100%**	0%	0%	0%	0%	0%	
Motilin MtlR	1	0	0	0	0	0	**1**	0	0	0	
(peptide, $G_{q/11}$, $G_{12/13}$)	100%	0%	0%	0%	0%	0%	**100%**	0%	0%	0%	
Melanin Conc. Hormone MCH2 R	1	0	0	0	**1**	0	0	0	0	0	
(hormone, $G_{q/11}$)	100%	0%	0%	0%	**100%**	0%	0%	0%	0%	0%	
Melanin Conc. Hormone MCH1 R	1	0	0	0	0	0	0	0	0	**1**	MCH1(T)
(hormone, G_s, $G_{i/o}$, $G_{q/11}$)	100%	0%	0%	0%	0%	0%	0%	0%	0%	**100%**	
Somatostatin SSTR3 R	1	0	0	0	0	0	0	**1**	0	0	R3(R) > R1(N)/2(N)
(hormone, $G_{i/o}$ > $G_{q/11}$)	100%	0%	0%	0%	0%	0%	0%	**100%**	0%	0%	/R4(N)/5(N) (G_i)
Somatostatin SSTR1/2/4/5 Rs	4	0	0	0	**4**	0	0	0	0	0	
(hormone, $G_{i/o}$)	100%	0%	0%	0%	**100%**	0%	0%	0%	0%	0%	
Glycoprotein Hormone FSH R	1	0	0	0	**1**	0	0	0	0	0	FSH(N) >
(hormone, G_s, $G_{i/o}$, $G_{q/11}$)	100%	0%	0%	0%	**100%**	0%	0%	0%	0%	0%	LH(T), TSH(A) (G_s)
Glycoprotein Hormone LHCG/TSH Rs	2	0	0	0	0	0	0	0	0	2	LH(T), TSH(A)
(hormone, G_s > $G_{q/11}$)	100%	0%	0%	0%	0%	0%	0%	0%	0%	100%	
Opioid δ/κ/μOpioid/ORL1 Rs	4	0	0	0	**4**	0	0	0	0	0	
(opioid, $G_{i/o}$)	100%	0%	0%	0%	**100%**	0%	0%	0%	0%	0%	
Chemokine(C) XCR1	1	0	0	0	0	0	**1**	0	0	0	
(chemokine, $G_{i/o}$)	100%	0%	0%	0%	0%	0%	**100%**	0%	0%	0%	
Chemokine(CC) CCR1–10 Rs	10	0	0	0	0	0	6	4	0	0	R2(K)/4–8(K) >
(chemokine, $G_{i/o}$)	100%	0%	0%	0%	0%	0%	60%	40%	0%	0%	R1/3/9/10(R) (G_i)?
Chemokine(CXC) CXCR1–6 Rs	6	0	0	0	1	0	5	0	0	0	R2–6(K) > R1(N) (G_i)
(chemokine, $G_{i/o}$)	100%	0%	0%	0%	17%	0%	83%	0%	0%	0%	
Chemokine(CX3C) CX3CR1	1	0	0	0	0	0	**1**	0	0	0	
(chemokine, $G_{i/o}$)	100%	0%	0%	0%	0%	0%	**100%**	0%	0%	0%	
Atypical Chemokine ACKR1/3/4 Rs	3	0	1	0	1	0	0	0	0	1	ACKR1(Q) >
(chemokine, no signaling/$G_?$)	100%	0%	33%	0%	33%	0%	0%	0%	0%	33%	R3(N),R4(S) ? ($G_?$)
Atypical Chemokine ACKR2	1	0	0	0	0	0	**1**	0	0	0	
(chemokine, arrestin)	100%	0%	0%	0%	0%	0%	**100%**	0%	0%	0%	
total number of Rs	86	total number of subgroups									31
R rate in the largest subgroups: 75/86	87%	rate of conserved subgroups for the 2nd aa of helix 8: 24/31									77%

5-TH, 5-hydroxytrypamine; FSH, follicle-stimulating hormone; LH, luteinizing hormone; TSH, thyroid-stimulating hormone; MC, acetylcholine (muscarinic) receptor.

Table S1. Classification of class A GPCRs by helix 8-2nd residues & subtypes of G proteins (continued).

GPCRs	Helix-8 Second Residue										Predicted Hierarchy,
(signal, G protein subtypes)	all	Glu	Gln	Asp	Asn	His	Lys	Arg	Trp	misc	the 2nd residue, misc.
Angiotensin II R1	1	0	0	0	0	0	**1**	0	0	0	R1(K) > R2(R) (G_i) ?
(hormone, $G_{i/o}$, $G_{q/11}$)	100%	0%	0%	0%	0%	0%	**100%**	0%	0%	0%	
Angiotensin II R2	1	0	0	0	0	0	0	**1**	0	0	
(hormone, $G_{i/o}$)	100%	0%	0%	0%	0%	0%	0%	**100%**	0%	0%	
$GnRH_1$ R	1	0	0	0	0	0	0	0	0	0	no helix 8
(hormone, $G_{q/11}$ > $G_{i/o}$)	100%	0%	0%	0%	0%	0%	0%	0%	0%	0%	
$GnRH_2$ R	1	0	0	0	0	0	0	0	0	0	no NPxxY, no helix 8
(hormone, ?)	100%	0%	0%	0%	0%	0%	0%	0%	0%	0%	
Apelin R	1	0	0	0	0	0	0	**1**	0	0	AGTL1
(peptide, $G_{i/o}$)	100%	0%	0%	0%	0%	0%	0%	**100%**	0%	0%	
Cholecystokinin $CCK_{1/2}$ Rs	2	0	0	0	0	0	0	**2**	0	0	G_q > G_s
(peptide, $G_{q/11}$)	100%	0%	0%	0%	0%	0%	0%	**100%**	0%	0%	
Bradykinin 2 R	1	0	0	0	0	0	0	**1**	0	0	R2(R) > R1(L) (G_i)
(peptide chemokine, G_s, $G_{i/o}$, $G_{q/11}$)	100%	0%	0%	0%	0%	0%	0%	**100%**	0%	0%	
Bradykinin 1 R	1	0	0	0	0	0	0	0	0	**1**	R1(L)
(peptide chemokine, $G_{i/o}$, $G_{q/11}$)	100%	0%	0%	0%	0%	0%	0%	0%	0%	**100%**	
Galanin 2 R	1	0	0	0	0	**1**	0	0	0	0	
(peptide hormone, $G_{q/11}$)	100%	0%	0%	0%	0%	**100%**	0%	0%	0%	0%	
Galanin 1/3 Rs	2	0	0	0	1	1	0	0	0	0	R3(H) > R1(N) (G_i)
(peptide hormone, $G_{i/o}$)	100%	0%	0%	0%	50%	50%	0%	0%	0%	0%	
Vassopressin V2 R	1	0	0	0	0	0	0	0	0	**1**	V2(S)
(hormone, G_s)	100%	0%	0%	0%	0%	0%	0%	0%	0%	**100%**	
Vassopressin V1a/b & Oxytocin OXT Rs	3	0	0	0	0	**3**	0	0	0	0	
(hormone, $G_{q/11}$)	100%	0%	0%	0%	0%	**100%**	0%	0%	0%	0%	
Cannabinoid CNR1/2 Rs	2	1	0	1	0	0	0	0	0	0	R1(D) > R2(E) (G_i)
(neurotransmitter/lipid, $G_{i/o}$ > G_s)	100%	50%	0%	50%	0%	0%	0%	0%	0%	0%	R2(E) > R1(D) (G_s)
GPER1 (GPR30)	1	0	0	0	0	0	0	0	0	**1**	R1(T)
(hormone, G_s, $G_{i/o}$, $G_{q/11}$)	100%	0%	0%	0%	0%	0%	0%	0%	0%	**100%**	
Adenosine $A_{2A/B}$ Rs	2	1	0	1	0	0	0	0	0	0	A_{2A}(E) > A_{2B}(D) (G_s)
(neurotransmitter, G_s)	100%	50%	0%	50%	0%	0%	0%	0%	0%	0%	
Adenosine $A_{1/3}$ Rs	2	0	0	0	0	0	**2**	0	0	0	
(neurotransmitter, $G_{i/o}$)	100%	0%	0%	0%	0%	0%	**100%**	0%	0%	0%	
Hydrocarboxyl acid $HCA_{1/2/3}$ Rs	3	0	0	0	0	0	0	0	0	**3**	R1/2/3(S), DPxxY-
(organic acid, $G_{i/o}$)	100%	0%	0%	0%	0%	0%	0%	0%	0%	**100%**	motif
Kisspeptin R	1	0	0	0	0	**1**	0	0	0	0	
(neuropeptide, $G_{q/11}$)	100%	0%	0%	0%	0%	**100%**	0%	0%	0%	0%	
Rexaxin/insulin-like family peptide 1/2 Rs	2	0	0	0	0	0	0	0	0	2	R1(P) > R2(F) (G_s)
(peptide hormones, G_s, $G_{i/o}$)	100%	0%	0%	0%	0%	0%	0%	0%	0%	100%	
Rexaxin/insulin-like family peptide 3/4 Rs	2	**2**	0	0	0	0	0	0	0	0	
(peptide hormones, $G_{i/o}$)	100%	**100%**	0%	0%	0%	0%	0%	0%	0%	0%	
Hypocretin (orexin) $OX_{1/2}$ Rs	2	0	0	0	0	0	**2**	0	0	0	
(peptide hormones, G_s, $G_{i/o}$, $G_{q/11}$)	100%	0%	0%	0%	0%	0%	**100%**	0%	0%	0%	
Bombesin BRS3/NMB/GRP Rs	3	0	0	0	0	0	0	0	0	**3**	(S, S, S)
(peptide, $G_{q/11}$)	100%	0%	0%	0%	0%	0%	0%	0%	0%	**100%**	
Endothelin A R	1	0	0	0	0	0	**1**	0	0	0	
(peptide, $G_{q/11}$)	100%	0%	0%	0%	0%	0%	**100%**	0%	0%	0%	
Endothelin B R	1	0	0	0	0	0	0	**1**	0	0	
(peptide, G_s)	100%	0%	0%	0%	0%	0%	0%	**100%**	0%	0%	
Neurotensin NTS_1 R	1	0	0	0	**1**	0	0	0	0	0	R1(N) > R2(S) (G_q)
(neuropeptide, $G_{q/11}$ > G_s/$G_{i/o}$)	100%	0%	0%	0%	**100%**	0%	0%	0%	0%	0%	
Neurotensin NTS_2 R	1	0	0	0	0	0	0	0	0	**1**	R2(S)
(neuropeptide, $G_{q/11}$)	100%	0%	0%	0%	0%	0%	0%	0%	0%	**100%**	
Neuromedin U NMU1/2 Rs	2	0	0	0	0	0	0	**2**	0	0	
(neuropeptide, $G_{q/11}$)	100%	0%	0%	0%	0%	0%	0%	**100%**	0%	0%	
Neuropeptide W/B $NPBW_{1/2}$ Rs	2	0	0	0	1	0	0	0	0	1	R1(N) > R2(S) (G_i)
(neuropeptide, $G_{i/o}$)	100%	0%	0%	0%	50%	0%	0%	0%	0%	50%	
Neuropeptides FF NPFF1/2 Rs	2	0	0	0	**2**	0	0	0	0	0	
(neuropeptide, $G_{i/o}$)	100%	0%	0%	0%	**100%**	0%	0%	0%	0%	0%	
Neuropeptides S NPS R	1	0	0	0	0	0	0	0	0	**1**	R(S)
(neuropeptide, $G_{q/11}$ > G_s)	100%	0%	0%	0%	0%	0%	0%	0%	0%	**100%**	
Neuropeptides Y 2/4 Rs	2	0	0	0	**2**	0	0	0	0	0	R1(N)/2(N)/4(N) >
(neuropeptide, $G_{i/o}$ > $G_{q/11}$)	100%	0%	0%	0%	**100%**	0%	0%	0%	0%	0%	R5(G) (G_i)
Neuropeptides Y 1/5 Rs	2	0	0	0	1	0	0	0	0	1	R1(N) > R5(G) (G_i)
(neuropeptide, $G_{i/o}$)	100%	0%	0%	0%	50%	0%	0%	0%	0%	50%	
total number of Rs	51	total number of subgroups									32
R rate in the largest subgroups: 43/51	84%	rate of conserved subgroups for the 2nd aa of helix 8: 24/32									75%

GnRH, gonadotropin-releasing hormone; AGTL1, angiotensin II receptor-like 1; GPER1, G protein-coupled estrogen receptor 1; BRS3, bombesin receptor subtype 3; NMBR, neuromedin B receptor; GRPR, gastrin releasing peptide receptor.

Table S1. Classification of class A GPCRs by helix 8-second residues & subtypes of G proteins (continued).

GPCRs	Helix-8 Second Residue										Predicted Hierarchy,
(signal, G protein subtypes)	all	Glu	Gln	Asp	Asn	His	Lys	Arg	Trp	misc	the 2nd residue, misc.
Proteinase-activated PAR 1/2/4 Rs	3	2	0	1	0	0	0	0	0	0	R1/4(E) > R2(D) (G_q),
(peptide, $G_{i/o}$, $G_{q/11}$)	100%	67%	0%	33%	0%	0%	0%	0%	0%	0%	DPxxY-motif
Proteinase-activated PAR3 R	1	0	0	0	0	0	0	0	0	**1**	R(T)
(peptide, ?)	100%	0%	0%	0%	0%	0%	0%	0%	0%	**100%**	
Ghrelin GHSR	1	0	0	0	0	0	**1**	0	0	0	
(peptide hormone, $G_{q/11}$ > $G_{i/o}$)	100%	0%	0%	0%	0%	0%	**100%**	0%	0%	0%	
Bile acid GPBA R	1	0	0	0	0	0	0	**1**	0	0	
(steroid, G_s)	100%	0%	0%	0%	0%	0%	0%	**100%**	0%	0%	
Formyl Peptide L2 R	1	0	0	0	**1**	0	0	0	0	0	L2(N) > 1(D)/L1(D)
(peptide, $G_{i/o}$, $G_{q/11}$)	100%	0%	0%	0%	**100%**	0%	0%	0%	0%	0%	(G_i)
Formyl peptide 1/L1 Rs	2	0	0	**2**	0	0	0	0	0	0	
(peptide, $G_{i/o}$)	100%	0%	0%	**100%**	0%	0%	0%	0%	0%	0%	
Complement Peptide C5AR1	1	0	0	0	0	0	0	0	0	**1**	C5AR1(G) (G_i > Gq)
(peptide, $G_{i/o}$ > $G_{q/11}$)	100%	0%	0%	0%	0%	0%	0%	0%	0%	**100%**	
Complement Peptide C3AR1	1	0	0	**1**	0	0	0	0	0	0	
(peptide, $G_?$)	100%	0%	0%	**100%**	0%	0%	0%	0%	0%	0%	
Complement Peptide C5AR2	1	0	0	0	0	0	0	0	0	**1**	C5AR2(A)
(peptide, Arrestin)	100%	0%	0%	0%	0%	0%	0%	0%	0%	**100%**	
Tachykinin $NK_{1/2}$ Rs	2	0	0	0	0	0	0	**2**	0	0	
(peptide, G_s, $G_{q/11}$)	100%	0%	0%	0%	0%	0%	0%	**100%**	0%	0%	
Tachykinin NK_3 R	1	0	0	0	0	0	0	**1**	0	0	
(peptide, $G_{q/11}$)	100%	0%	0%	0%	0%	0%	0%	**100%**	0%	0%	
Prolactin-releasing peptide R	1	0	0	0	0	0	0	0	0	**1**	R(S)
(peptide, $G_{q/11}$)	100%	0%	0%	0%	0%	0%	0%	0%	0%	**100%**	
QRFP R	1	0	0	0	**1**	0	0	0	0	0	
(peptide, $G_{i/o}$, $G_{q/11}$)	100%	0%	0%	0%	**100%**	0%	0%	0%	0%	0%	
Prokineticin PKR1/2 Rs	2	0	0	0	0	0	0	0	0	**2**	R1/2(T), NTxxF-motif
(peptide, $G_{q/11}$ > G_s)	100%	0%	0%	0%	0%	0%	0%	0%	0%	**100%**	
Urotencin-Ⅱ R	1	0	0	0	**1**	0	0	0	0	0	
(peptide, $G_{q/11}$)	100%	0%	0%	0%	**100%**	0%	0%	0%	0%	0%	
Oxoglutamate OXGR1 R	1	0	0	0	**1**	0	0	0	0	0	
(organic acid, $G_{q/11}$)	100%	0%	0%	0%	**100%**	0%	0%	0%	0%	0%	
Succinate R	1	0	0	0	0	**1**	0	0	0	0	
(organic acid, $G_{i/o}$, $G_{q/11}$)	100%	0%	0%	0%	0%	**100%**	0%	0%	0%	0%	
Purinergic P2Y1 R	1	0	0	0	0	0	0	0	0	**1**	R1(T), DPxxY-motif
(nucleotide, $G_{q/11}$ > $G_{i/o}$)	100%	0%	0%	0%	0%	0%	0%	0%	0%	**100%**	
Purinergic P2Y2 R	1	0	0	0	0	0	0	**1**	0	0	
(nucleotide, $G_{q/11}$ > $G_{i/o}$/G_{12})	100%	0%	0%	0%	0%	0%	0%	**100%**	0%	0%	
Purinergic P2Y4 R	1	0	0	0	0	0	**1**	0	0	0	(D/H)PxxY-motif
(nucleotide, $G_{q/11}$)	100%	0%	0%	0%	0%	0%	**100%**	0%	0%	0%	
Purinergic P2Y6 R	1	0	0	0	0	0	**1**	0	0	0	
(nucleotide, $G_{q/11}$ > G_{12})	100%	0%	0%	0%	0%	0%	**100%**	0%	0%	0%	
Purinergic P2Y12/13/14 Rs	3	0	0	0	0	0	1	0	0	2	R13(K) >
(nucleotide, $G_{i/o}$)	100%	0%	0%	0%	0%	0%	33%	0%	0%	67%	R14(P), R12(S)
Prostaglandin D DR1 R	1	0	0	0	0	0	0	0	0	**1**	R(P), (N/D)PWxF-
(prostanoid, G_s)	100%	0%	0%	0%	0%	0%	0%	0%	0%	**100%**	motif
Prostaglandin D DR2 R	1	0	0	**1**	0	0	0	0	0	0	(N/D)PWxF-motif
(prostanoid, $G_{i/o}$)	100%	0%	0%	**100%**	0%	0%	0%	0%	0%	0%	
Prostaglandin E2 EP2 R	1	0	0	0	0	0	0	0	0	**1**	R(P), DPWxY-motif
(prostanoid, G_s)	100%	0%	0%	0%	0%	0%	0%	0%	0%	**100%**	
Prostaglandin E2 EP4 R	1	0	0	0	0	0	0	0	0	**1**	R(T), DPWxY-motif
(prostanoid, G_s > $G_{i/o}$)	100%	0%	0%	0%	0%	0%	0%	0%	0%	**100%**	
Prostaglandin E2 EP1 R	1	0	0	0	0	0	0	0	0	**1**	R(A), DPWxY-motif
(prostanoid, $G_{q/11}$)	100%	0%	0%	0%	0%	0%	0%	0%	0%	**100%**	
Prostaglandin E2 EP3 R	1	0	0	0	0	0	0	0	0	**1**	R(I), DPWxY-motif
(prostanoid, $G_{i/o}$)	100%	0%	0%	0%	0%	0%	0%	0%	0%	**100%**	
Prostaglandin F FP R	1	0	0	0	0	0	0	0	0	**1**	R(A), DPWxY-motif
(prostanoid, $G_{q/11}$)	100%	0%	0%	0%	0%	0%	0%	0%	0%	**100%**	
Prostaglandin I2 IP R	1	0	0	0	0	0	0	0	0	**1**	R(A), DPWxF-motif
(prostanoid, G_s > $G_{i/o}$)	100%	0%	0%	0%	0%	0%	0%	0%	0%	**100%**	
Thromboxane A2 TP R	1	0	0	0	0	0	0	0	0	**1**	R(A), DPWxY-motif
(prostanoid, $G_{q/11}$)	100%	0%	0%	0%	0%	0%	0%	0%	0%	**100%**	
total number of Rs	38	total number of subgroups									31
R rate in the largest subgroups: 35/38	92%	rate of conserved subgroups for the 2nd aa of helix 8: 29/31									94%

GHSR, growth hormone secretagogue receptor type 1; C3AR1, complement C3a receptor 1; C5AR1, complement C5a receptor 1; QRFP, proglutamated RFamide peptide; PTGDR, prostaglandin D2 receptor; PTGER1/2/3/4, prostaglandin E receptor 1/2/3/4; PTGFR, prostaglandin F receptor; PTGIR, prostaglandin I2 receptor; TBXA2R, thromboxane A2 receptor.

Table S1. Classification of class A GPCRs by helix 8-second residues & subtypes of G proteins (continued).

GPCRs	Helix-8 Second Residue										Predicted Hierarchy,
(signal, G protein subtypes)	all	Glu	Gln	Asp	Asn	His	Lys	Arg	Trp	misc	the 2nd residue, misc.
Oxoeicosanoid R	1	0	0	0	**1**	0	0	0	0	0	DPxxY-motif
(leukotriene, $G_{i/o}$)	100%	0%	0%	0%	**100%**	0%	0%	0%	0%	0%	
Cysteinyl leukotriene 1/2 Rs	2	0	0	0	**2**	0	0	0	0	0	(N/D)PxxY-motif
(leukotriene, $G_{q/11} > G_{i/o}$)	100%	0%	0%	0%	**100%**	0%	0%	0%	0%	0%	
Leukotriene B4 BLT_1 R	1	0	0	**1**	0	0	0	0	0	0	R2(D) > R(G) (G_i),
(leukotriene, $G_{i/o} > G_{q/11}$)	100%	0%	0%	**100%**	0%	0%	0%	0%	0%	0%	NPxxY-motif
Leukotriene B4 BLT_1 R	1	0	0	0	0	0	0	0	0	**1**	R(G), NPxxY-motif
(leukotriene, $G_{i/o}$, $G_{q/11}$)	100%	0%	0%	0%	0%	0%	0%	0%	0%	**100%**	
Chemerin CMKLR1	1	0	0	**1**	0	0	0	0	0	0	
(adipokine, $G_{i/o}$)	100%	0%	0%	**100%**	0%	0%	0%	0%	0%	0%	
Chemerin GPR1	1	0	0	0	0	0	**1**	0	0	0	
(adipokine, ?)	100%	0%	0%	0%	0%	0%	**100%**	0%	0%	0%	
CCRL2 R	1	0	0	0	0	0	0	0	0	**1**	R(T)
(chemokine, $G_?$)	100%	0%	0%	0%	0%	0%	0%	0%	0%	**100%**	
LPAR1/2	2	**2**	0	0	0	0	0	0	0	0	(N/D)PxxY-motif
(lipid signal, $G_{i/o}$, $G_{q/11}$, G_{12})	100%	**100%**	0%	0%	0%	0%	0%	0%	0%	0%	
LPAR3	1	0	0	**1**	0	0	0	0	0	0	
(lipid signal, $G_{i/o}$, $G_{q/11}$)	100%	0%	0%	**100%**	0%	0%	0%	0%	0%	0%	
LPAR4	1	0	0	0	0	0	0	0	0	**1**	R4(S), DPxxY-motif
(lipid signal, G_s, $G_{i/o}$, $G_{q/11}$, G_{12})	100%	0%	0%	0%	0%	0%	0%	0%	0%	**100%**	
LPAR6	1	0	0	0	0	0	0	0	0	**1**	R6(T), DPxxY-motif
(lipid signal, G_s, $G_{i/o}$, G_{12})	100%	0%	0%	0%	0%	0%	0%	0%	0%	**100%**	
LPAR5	1	0	0	0	0	0	0	0	0	**1**	R5(G), DPxxY-motif
(lipid signal, $G_{q/11}$, G_{12})	100%	0%	0%	0%	0%	0%	0%	0%	0%	**100%**	
S1PR1	1	**1**	0	0	0	0	0	0	0	0	
(lipid mediator, $G_{i/o}$)	100%	**100%**	0%	0%	0%	0%	0%	0%	0%	0%	
S1PR3	1	**1**	0	0	0	0	0	0	0	0	
(lipid mediator, $G_{i/o}$, $G_{q/11}$, $G_{12/13}$)	100%	**100%**	0%	0%	0%	0%	0%	0%	0%	0%	
S1PR4/5	2	1	0	1	0	0	0	0	0	0	R4(E) = R5(D) (G_i)
(lipid mediator, $G_{i/o}$, $G_{12/13}$)	100%	50%	0%	50%	0%	0%	0%	0%	0%	0%	
S1PR2	1	0	0	**1**	0	0	0	0	0	0	
(lipid mediator, G_s, $G_{q/11}$, $G_{12/13}$)	100%	0%	0%	**100%**	0%	0%	0%	0%	0%	0%	
Prokineticin PKR1/2 Rs	2	0	0	0	0	0	0	0	0	**2**	R1/2(T), NTxxF-motif,
(protein, $G_{q/11} > G_s$)	100%	0%	0%	0%	0%	0%	0%	0%	0%	**100%**	8th from F
Platelet-activating factor R	1	0	0	0	0	0	**1**	0	0	0	DPxxY-motif
(lipid, $G_{i/o}$, $G_{q/11}$)	100%	0%	0%	0%	0%	0%	**100%**	0%	0%	0%	
Free fatty acid FFAR1/2/4 Rs	3	1	0	0	0	0	0	1	0	1	R1(G)/2(V)/4(E),
(lipid, $G_{q/11}$)	100%	33%	0%	0%	0%	0%	0%	33%	0%	33%	(N/D)PxxY-motif
Free fatty acid FFAR3	1	0	0	0	0	0	0	0	0	**1**	R3(G), (N/D)PxxY-
(lipid, $G_{i/o}$)	100%	0%	0%	0%	0%	0%	0%	0%	0%	**100%**	motif
GPR18 R	1	0	**1**	0	0	0	0	0	0	0	DVxxY-motif
(N-arachidonoylglycine, $G_{i/o}$, $G_{q/11}$)	100%	0%	**100%**	0%	0%	0%	0%	0%	0%	0%	
GPR119 R	1	**1**	0	0	0	0	0	0	0	0	NPxxY-motif
(N-oleoylethanolamide, G_s)	100%	**100%**	0%	0%	0%	0%	0%	0%	0%	0%	
GPR55 R	1	**1**	0	0	0	0	0	0	0	0	DVxxY-motif
(lysophosphatidylinositol, $G_{q/11}$, $G_{12/13}$)	100%	**100%**	0%	0%	0%	0%	0%	0%	0%	0%	
total number of Rs	29	total number of subgroups									23
R rate in the largest subgroups: 26/29	90%	rate of conserved subgroups for the 2nd aa of helix 8: 21/23									91%

CMKLR1, chemerin chemokine-like receptor 1; CCLR2, chemokine (C-C) receptor-like receptor2; LPAR, lysophosphatidic acid receptor.

Human GPCRs *mOR-S6*	*TM7-NPxxY helix 8*	Human GPCRs *mOR-S6*	*TM7-NPxxY helix 8*	Subclass.G-pr_subtypes
Rhod(312)	NPVIYIMMNK**Q**FRNCMLTTIC	OPN1SW(309)	NPIIYCFMNK**Q**FQACIMKMVC	Rhod/O.G_t
OPN1MW(328)	NPVIYVFMNR**Q**FRNCILQLFG	OPN1LW(328)	NPVIYVFMNR**Q**FRNCILQLFG	Rhod/O.G_t
OPN4(356)	NPIIYAITHP**K**YRVAIAQHLP	OPN5(312)	NPIIYQVIDY**K**FACCQTGGLK	Rhod/O.G_i/(G_q)
OPN3(315)	NPVIYVFMIR**K**FRRSLLQLLC			Rhodopsin/Opsin.G_i?
β_1AdR(382)	NPIIYC.RSP**D**FRKAFQGLLC	β_2AdR(331)	NPLIYC.RSP**D**FRIAFQELLC	AdR.G_s
β_3AdR(351)	NPLIYC.RSP**D**FRSAFRRLLC			Adrenergic R.G_s
α_{1A}AdR(332)	NPIIYPCSSQ**E**FKKAFQNVLR	α_{1B}AdR(354)	NPIIYPCSSK**E**FKRAFVRTLG	AdR.G_q
α_{1D}AdR(408)	NPLIYPCSSR**E**FRRAFLRLLC			Adrenergic R.G_q
α_{2A}AdR(432)	NPVIYTIFNH**D**FRRAFKKILC	α_{2B}AdR(429)	NPVIYTIFNQ**D**FRRAFRRILC	AdR.G_i
α_{2C} AdR(443)	NPVIYTVFNQ**D**FRRSFKHILF			Adrenergic R.G_i
D1(336)	NPIIYAF.NA**D**FRKAFSTLLG	D5(365)	NPVIYAF.NA**D**FQKVFAQLLG	DopR.G_s
D2(432)	NPIIYTTFNI**E**FRKAFLKILH	D3(389)	NPVIYTTFNI**E**FRKAFLKILS	DopR.G_i
D4(454)	NPVIYTVFNA**E**FRNVFRKALR			Dopamine R.G_i
5-HT$_6$(326)	NPIIYPLFMR**D**FKRALGRFLP	5-HT$_7$(390)	NPFIYAFFNR**D**LRTTYRSLLQ	SeroR.G_s
5-HT$_4$(318)	NPFLYAFLNK**S**FRRAFLIILC			Serotonin R.G_s
5-HT$_{2A}$(386)	NPLVYTLFNK**T**YRSAFSRYIQ	5-HT$_{2C}$(374)	NPLVYTLFNK**I**YRRAFSNYLR	SeroR.G_q>G_i
5-HT$_{2B}$(386)	NPLVYTLFNK**T**FRDAFGRYIT			Serotonin R.G_q
5-HT$_{1D}$(362)	NPIIYTVFNE**E**FRQAFQKIVP	5-HT$_{5A}$(344)	NPLIYTAFNK**N**YNSAFKNFFS	SeroR.G_i
5-HT$_{1A}$(406)	NPVIYAYFNK**D**FQNAFKKIIK	5-HT$_{1B}$(375)	NPIIYTMSNE**D**FKQAFHKLIR	SeroR.G_i
5-HT$_{1E}$(350)	NPLLYTSFNE**D**FKLAFKKLIR	5-HT$_{1F}$(353)	NPLIYTIFNE**D**FKKAFQKLVR	SeroR.G_i
H1(474)	NPLIYPLCNE**N**FKKTFKRILH			Histamine R.G_q
H2(294)	NPILYAALNR**D**FRTGYQQLFC			Histamine R.G_q>G_s
H4(364)	NPLLYPLCHK**R**FQKAFLKIFC	H3(418)	NPVLYPLCHH**S**FRRAFTKLLC	HistR.G_i
MC1(304)	DPLIYAFHSQ**E**LRRTLKEVLT	MC2(282)	DPFIYAFRSP**E**LRDAFKKMIF	MelaR.G_s
MC3(305)	DPLIYAFRSL**E**LRNTFREILC	MC4(308)	DPLIYALRSQ**E**LRKTFKEIIC	MelaR.G_s
MC5(301)	DPLIYAFRSQ**E**MRKTFKEIIC			Melanocortin R.G_s
MTNR1A(301)	NAIIYGLLNQ**N**FRKEYRRIIV	MTNR1B(313)	NAIVYGLLNQ**N**FRREYKRILL	MelatR.G_i
MtlR(361)	NPILYNLISK**K**YRAAAFKLLL			Motilin R.G_q/G_{12}
MCH1(386)	NPFVYIVLCE**T**FRKRLVLSVK			Melanin Concentration Hormone R.G_s/G_i/G_q
MCH2(312)	NPFLYILLSG**N**FQKRLPQIQR			Melanin Concentration Hormone R.G_q
SSTR3(319)	NPILYGFLSY**R**FKQGFRRVLL			Somatstatin R.G_i>G_q
SSTR1(329)	NPILYGFLSD**N**FKRSFQRILC	SSTR2(318)	NPILYAFLSD**N**FKKSFQNVLC	SomaR.G_i
SSTR4(317)	NPILYGFLSD**N**FRRFFQRVLC	SSTR5(310)	NPVLYGFLSD**N**FRQSFQKVLC	SomaR.G_i
FSHR(632)	NPFLYAIFTK**N**FRRDFFILLS			Glycoprotein hormone R.G_s/G_i/G_q
LHCGR(629)	NPFLYAIFTK**T**FQRDFFLLLS	TSHR(684)	NPFLYAIFTK**A**FQRDVFILLS	GlyHR.G_s>G_q
δOpioid(324)	NPVLYAFLDE**N**FKRCFRQLCR	κOpioid(336)	NPILYAFLDE**N**FKRCFRDFCF	OpioR.G_i
μOpioid(344)	NPVLYAFLDE**N**FKRCFREFCI	ORL1(325)	NPILYAFLDE**N**FKACFRKFCC	OpioR.G_i
XCR1(293)	NPVLYVFVGV**K**FRTHLKHVLR			Chemokine(C) R.G_i
CCR2(311)	NPIIYAFVGE**K**FRSLFHIALG	CCR4(310)	NPIIYFFLGE**K**FRKYILQLFK	Chem(C-C)R.G_i
CCR5(303)	NPIIYAFVGE**K**FRNYLLVFFQ	CCR6(322)	NPVLYAFIGQ**K**FRNYFLKILK	Chem(C-C)R.G_i
CCR7(332)	NPFLYAFIGV**K**FRNDLFKLFK	CCR8(306)	NPVIYAFVGE**K**FKKHLSEIFQ	Chem(C-C)R.G_i
CCR1(307)	NPVIYAFVGE**R**FRKYLRQLFH	CCR3(307)	NPVIYAFVGE**R**FRKYLRHFFH	Chem(C-C)R.G_i
CCR9(323)	NPVLYVFVGE**R**FRRDLVKTLK	CCR10(316)	NPVLYAFLGL**R**FRQDLRRLLR	Chem(C-C)R.G_i
CXCR2(320)	NPLIYAFIGQ**K**FRHGLLKILA	CXCR3(324)	NPLLYAFVGV**K**FRERMWMLLL	Chem(CXC)R.G_i
CXCR6(294)	NPVLYAFVSL**K**FRKNFWKLVK	CXCR5(328)	NPMLYTFAGV**K**FRSDLSRLLT	Chem(CXC)R.G_i
CXCR4(308)	NPILYAFLGA**K**FKTSAQHALT	CXCR1(311)	NPIIYAFIGQ**N**FRHGFLKILA	Chem(CXC)R.G_i
CX3CR1(299)	NPLIYAFAGE**K**FRRYLYHLYG			Chemokine(CX3C) R.G_i
ACKR2(318)	SPILYAFSSH**R**FRQYLKAFLA			Atypical chemokine R.Arrestin
ACKR4(309)	NPILYVFMGA**S**FKNYVMKVAR			Atypical Chemokine R.?
ACKR3(321)	NPVLYSFINR**N**YRYELMKAFI	ACKR1(310)	TPLLLALFCH**Q**ATRTLLPSLP	AtChem R.?

Supplementary Fig. S1. Alignment of amino acid sequences of NPxxY motif and helix 8 of class A GPCRs. The 204 human non-olfactory GPCRs are shown. The **bold residue** indicates the 2nd one of helix 8, the position of which in the amino acid sequence is shown in the parentheses. Helix 8 was expected to be formed by hydrophobic residues at the 3rd and more than two of the 7th, 8th, 10th, and 11th positions. The conserved TM7 motif is (N/D)Pxx(Y/F). 5-TH, 5-hydroxytrypamine; ACM, acetylcholine (muscarinic); FSH, follicle-stimulating hormone; LH, luteinizing hormone; TSH, thyroid-stimulating hormone.

Human GPCRs		Human GPCRs		Subclass.G-pr_subtypes
mOR-S6	*TM7-NPxxY helix 8*	*mOR-S6*	*TM7-NPxxY helix 8*	
ACM$_2$(446)	NPACYALCNA**T**FKKTFKHLLM	ACM$_4$(459)	NPACYALCNA**T**FKKTFRHLLL	AcM R.G_i
ACM$_1$(424)	NPMCYALCNK**A**FRDTFRLLLL	ACM$_3$(550)	NPVCYALCNK**T**FRTTFKMLLL	AcM R.G_q
ACM$_5$(501)	NPICYALCNR**T**FRKTFKMLLL			Acetylcholine(muscarinic) R.G_q
AGT1R(308)	NPLFYGFLGK**K**FKRYFLQLLK			Angiotensin II R.G_q/G_i/G_{12}
AGT2R(324)	NPFLYCFVGN**R**FQQKLRSVFR			AngIIR.$G_{i/o}$
GnRH$_1$	DPLIYGYFSL			Gonadotrophin-releasing hormone R.G_q>G_i
GnRH$_2$	no helix 8			Gonadotrophin-releasing hormone R.?
APLN(315)	NPFLYAFFDP**R**FRQACTSMLC			Apelin R(Angiotensin Receptor-like 1).$G_{i/o}$
CCK1(376)	NPIIYCFMNK**R**FRLGFMATFP	CCK2(396)	NPLVYCFMHR**R**FRQACLETCA	Cholcy.k.R.G_q
BDKRB2(338)	NPLVYVIVGK**R**FRKKSWEVYQ			Bradykinin R.G_s/G_i/G_q
BDKRB1(318)	NPVIYVFVGR**L**FRTKVWELYK			Bradykinin R.G_i/G_q
GALR2(298)	NPIVYALVSK**H**FRKGFRTICA			Galanin R.G_q
GALR3(297)	NPLVYALASR**H**FRARFRRLWP	GALR1(309)	NPIIYAFLSE**N**FRKAYKQVFK	GalR.G_i
V2(331)	NPWIYASFSS**S**VSSELRSLLC			Vasopressin R.G_s
V1a(354)	NPWIYMFFSG**H**LLQDCVQSFP	V1b(344)	NPWIYMGFNS**H**LLPRPLRHLA	VassR.G_q
OXTR(335)	NPWIYMLFTG**H**LFHELVQRFL			Oxytocin R.G_q
CNR1(403)	NPIIYALRSK**D**LRHAFRSMFP	CNR2(305)	NPVIYALRSG**E**IRSSAHHCLA	CannR.G_i>G_s
GPER1(320)	NPLIYSFLGE**T**FRDKLRLYIE			GP Estrogen R1.G_s
A$_{2A}$(294)	NPFIYAYRIR**E**FRQTFRKIIR	A$_{2B}$(296)	NPIVYAYRNR**D**FRYTFHKIIS	AdenR.G_s
A$_3$(288)	NPIVYAYKIK**K**FKETYLLILK	A$_1$(294)	NPIVYAFRIQ**K**FRVTFLKIWN	AdenR.G_i
HCA1(284)	DPLVYYFSSP**S**FPKFYNKLKI	HCA2(300)	DPVVYYFSSP**S**FPNFFSTLIN	HCA R.G_s
HCA3(300)	DPVVYYFSSP**S**FPNFFSTLIN			Hydrocarboxyl acid R.G_i
KISSR(329)	NPLLYAFLGS**H**FRQAFRRVCP			Kisspeptin R.G_q
RXFP1(687)	NPILYTLTTR**P**FKEMIHRFWY	RXFP2(697)	NPILYTLTTN**F**FKDKLKQLLH	RelaxR.G_s/G_i
RXFP3(395)	NPVLYCLVRR**E**FRKALKSLLW	RXFP4(315)	NPVLYCLLRR**E**PRQALAGTFR	RelaxR.G_i
OX1(364)	NPIIYNFLSG**K**FREQFKAAFS	OX2(370)	NPIIYNFLSG**K**FREEFKAAFS	OrexnR.G_s/G_i/G_q
BRS3(336)	NPFALYWLSK**S**FQKHFKAQLF	NMBR(330)	NPFALYLLSE**S**FRRHFNSQLC	BombR.G_q
GRPR(328)	NPFALYLLSK**S**FRKQFNTQLL			Gastrin releasing peptide R.G_q
EDNRA(375)	NPIALYFVSK**K**FKNCFQSCLC			Endthelin R.G_q
EDNRB(392)	NPIALYLVSK**R**FKNCFKSCLC			Endthelin R.G_s/G_i/G_q
NMUR1(362)	NPVLYSLMSS**R**FRETFQEALC	NMUR2(332)	NPIIYNLLSR**R**FQAAFQNVIS	NeurM U R.G_q
NTS1(370)	NPILYNLVSA**N**FRHIFLATLA			Neurotensin R.G_q>G_s/G_i
NTS2(364)	TPLLYNAVSS**S**FRKLFLEAVS			Neurotensin R.G_q
NPBW2(322)	NPFLYAFLDD**N**FRKNFRSILR	NPBW1(313)	NPFLYAFLDA**S**FRRNLRQLIT	NeurP W/B R.G_i
NPFF1(335)	NPIIYGYFNE**N**FRRGFQAAFR	NPFF2(441)	NPIIYGFFNE**N**FRRGFQEAFQ	NeurP FF R.G_i
NPS(336)	NPLIYCVFSSSI**S**FPCRVIRL			Neuropeptides S R.G_q>G_s
NPY2(332)	NPLLYGWMNS**N**YRKAFLSAFR	NPY4(328)	NPFIYGFLNT**N**FKKEIKALVL	NeP Y R.G_i>G_q
NPY1(326)	NPIFYGFLNK**N**FQRDLQFFFN	NPY5(431)	NPILYGFLNN**G**IKADLVSLIH	NeP Y R.G_i
PAR1(377)	DPLIYYYASS**E**CQRYVYSILC	PAR2(350)	DPFVYYFVSH**D**FRDHAKNALL	PA R.G_i/G_q
PAR4(346)	DPFIYYYVSA**E**FRDKVRAGLF			Proteinase-activated R.G_i/G_q
PAR3(364)	DPFLYFLMSK**T**RNHSTAYLTK	no helix 8		Proteinase-activated R.?
GHSR(329)	NPILYNIMSK**K**YRVAVFRLLG			Ghrelin(Growth hormone secretagogue) R.G_q>G_i
GPBAR(286)	VPVAMGLGDQ**R**YTAPWRAAAQ			Bile acid R.G_s
FPR3(308)	NPILYVFMGR**N**FQERLIRSLP			Formyl peptide R.G_i>G_q
FPR1(307)	NPMLYVFMGQ**D**FRERLIHALP	FPR2(308)	NPMLYVFVGQ**D**FRERLIHSLP	FormP.G_i
C5a1(306)	NPIIYVVAGQ**G**FQGRLRKSLP			Complement Peptide R.$G_{i/o}$>G_q
C3a(441)	NPFLYALLGK**D**FRKKARQSIQ			Complement Peptide R.$G_?$
C5a2(297)	NPMLFLYFGR**A**QLRRSLPAAC			Complement Peptide R.Arrestin
NK1(311)	NPIIYCCLND**R**FRLGFKHAFR	NK2(313)	NPIIYCCLNH**R**FRSGFRLAFR	TachyR.G_s/G_q
NK3(362)	NPIIYCCLNK**R**FRAGFKRAFR			Tachykinin R.G_q

Supplementary Fig. S1. Alignment of amino acid sequences of NPxxY motif and helix 8 of class A GPCRs (continued). NPS may cause a shift in the position of helix 8 by two amino acids. GnRH, gonadotropin-releasing hormone; AGTL1, angiotensin II receptor-like 1; GPER1, G protein-coupled estrogen receptor 1; BRS3, bombesin receptor subtype 3; NMBR, neuromedin B receptor; ENDRA/B, endothelin receptor type A/B; NMUR, neuromedin U receptor; NTSR, neurotensin receptor; FPR, formyl peptide receptor; C3AR1, complement C3a receptor 1; C5AR1, complement C5a receptor 1.

Human GPCRs	*mOR-S6 TM7 NPxxY helix 8*	Human GPCRs	*mOR-S6 TM7 NPxxY helix 8*	Subclass.G-pr_subtypes
PrRPR(341)	NPFIYAWLHD**S**FREELRKLLV			Prolactin-releasing peptide R.G_q
QRFP(338)	NPIVYAFMNE**N**FKKNVLSAVC			Pyroglutamylated RFamide peptide R.G_i/G_q
PKR1(349)	NTLCFVTVKND**T**VKYFKKIML	PKR2(340)	NTVCFVTVKNN**T**MKYFKKMML	PKR.$G_q>G_s$
UR2R(321)	NPFLYTLLTR**N**YRDHLRGRVR			Urotensin R.G_q
OXGR1(308)	NLLLYVVVSD**N**FQQAVCSTVR			Oxoglutamete R.G_q
SucR(301)	NPVFYFLLGD**H**FRDMLMNQLR			Succinate R.G_i/G_q
P2RY1(330)	DPILYFLAGD**T**FRRRLSRATR			Purinergic P2Y R.$G_q>G_i$
P2RY2(312)	DPVLYFLAGQ**R**LVRFARDAKP			Purinergic P2Y R.$G_q>G_i/G_{12}$
P2RY4(312)	DPVLYLLTGD**K**YRRQLRQLCG			Purinergic P2Y R.G_q
P2RY6(307)	DPILFYFTQK**K**FRRRPHELLQ			Purinergic P2Y R.$G_q>G_s$
P2RY8(299)	DPFVYYFASR**E**FQLRLREYLG	P2RY10(311)	DPILYYFMAS**E**FRDQLSRHGS	P2Y R.?
P2RY11(327)	HPLLYMAAVP**S**LGCCCRHCPG			Purinergic P2Y R.$G_q>G_s$
P2RY12(304)	DPFIYFFLCK**S**FRNSLISMLK	P2RY13(322)	DPLIYIFLCK**K**FTEKLPCMQG	P2Y R.G_i
P2RY14(301)	DPIIYFFLCQ**P**FREILCKKLH			Purinergic P2Y P2Y R.G_i
DR1(329)	DPWIFIIFRS**P**VFRIFFHKIF			Prostagrandin D R.G_s
DR2(310)	NPVLYVLTCP**D**MLRKLRRSLR			Prostagrandin D R.G_i
ER3(352)	DPWVYLLLRK**I**LLRKFCQIRY			Prostagrandin E2 R.$G_i>G_q$
ER1(357)	DPWVYILLRQ**A**VLRQLLRLLP			Prostagrandin E2 R.$G_q>G_i$
ER2(321)	DPWVFAILRP**P**VLRLMRSVLC			Prostagrandin E2 R.G_s
ER4(335)	DPWIYILLRK**T**VLSKAIEKIK			Prostagrandin E2 R.$G_s>G_i$
FR(310)	DPWVYILLRK**A**VLKNLYKLAS			Prostagrandin F R.$G_q>G_s$
IR(298)	DPWVFILFRK**A**VFQRLKLWVC			Prostagrandin I2 R.$G_s>G_i/G_q$
TA2R(314)	DPWVYILFRR**A**VLRRLQPRLS			Thromboxane A2 R.G_q
OXER(361)	DPVLYCFSSP**N**FLHQSRALLG			Oxoeicosanoid R.G_i
CYSLTR1(301)	DPLLYFFSGG**N**FRKRLSTFRK	CYSLTR2(321)	NPLLYYFAGE**N**FKDRLKSALR	CysLeR.$G_q>G_i$
BLTR2(325)	NPVLYVFTAG**D**LLPRAGPRFL			Leukotriene B4 R.$G_{i/o}>G_q$
BLTR1(291)	NPVLYACAGG**G**LLRSAGVGFV			Leukotriene B4 R.G_i/G_q
CMKLR1(322)	NPILYVFMGQ**D**FKKFKVALFS			Chemerine Chemokine-like R1(Anaphylatoxin R).G_i
GPR1(310)	NPILYVLISK**K**FQARFRSSVA			Chemerine R.?
CCRL2(306)	NPLLYAFLDG**T**FSKYLCRCFH			Chemokine(C-C) R.like 2 R.?
LPAR1(317)	NPIIYSYRDK**E**MSATFRQILC	LPAR2(301)	NAAVYSCRDA**E**MRRTFRRLLC	LisAR.$G_i/G_q/G_{12}$
LPAR3(299)	NPIIYSYKDE**D**MYGTMKKMIC			Lisophosphatidic acid R.G_i/G_q
LPAR4(317)	DPFIYYFTLE**S**FQKSFYINAH			Lisophosphatidic acid R.$G_s/G_i/G_q/G_{12}$
LPAR6(297)	DPIVYYFTSD**T**IQNSIKMKNW			Lisophosphatidic acid R.$G_s/G_i/G_{12}$
LPAR5(303)	DPLVYYFSAE**G**FRNTLRGLGT			Lisophosphatidic acid R.G_q/G_{12}
S1PR1(317)	NPIIYTLTNK**E**MRRAFIRIMS			Sphingosine-1-phosphate R.G_i
S1PR2(294)	NPVIYTWRSR**D**LRREVLRPLQ			Sphingosine-1-phosphate R.$G_s/G_q/G_{12}$
S1PR3(304)	NPVIYTLASK**E**MRRAFFRLVC			Sphingosine-1-phosphate R.$G_i/G_q/G_{12}$
S1PR4(313)	NPIIYSFRSR**E**VCRAVLSFLC	S1PR5(312)	NPIIYTLTNR**D**LRHALLRLVC	SphPR.G_i/G_{12}
PAF(299)	DPVIYCFLTK**K**FRKHLTEKFY			Platelet-activating factor R.G_i/G_q
FFAR1(284)	NPLVTGYLGR**G**PGLKTVCAAR	FFAR2(279)	DPLLFYFSSS**V**VRRAFGRGLQ	FreeFAR.G_q
FFAR4(345?)	NPILYNMTLCRN**E**WKKIFCCF			Free fatty acid R.G_q
FFAR3(282)	DPFVYYFSSS**G**FQADFHELLR			Free fatty acid R.G_i
GPR18(292)	DVILYYIVSK**Q**FQARVISVML			N-arachidonoylglycine (lipid) R.G_i/G_q
GP119(285)	NPLIYAYWQK**E**VRLQLYHMAL			N-oleoylethanolamide (lipid) R.G_s
GPR55(294)	DVFCYYFVIK**E**FRMNIRAHRP			lysophosphatidylinositol(lipid) R.G_q/G_{12}

Supplementary Fig. S1. Alignment of amino acid sequences of NPxxY motif and helix 8 of class A GPCRs (continued). Some of their helical structures are likely to be unstable. PKR1/2 and FFAR4 may cause a shift in the position of helix 8 by one and two amino acids, respectively. PKR, prokineticin receptor; Suc, succinate; MTNR1A/B, melatonin receptor 1A/B; NPY1/2/4/5R, neuropeptide Y receptor Y1/2/4/5; QRFP, proglutamated RFamide peptide; CMKLR1, chemokine-like receptor 1; CCRL2, chemokine (C-C) receptor-like receptor 2; PTGDR, prostaglandin D2 receptor; PTGER1/2/3/4, prostaglandin E receptor 1/2/3/4; PTGFR, prostaglandin F receptor; PTGIR, prostaglandin I2 receptor; TBXA2R, thromboxane A2 receptor; CYSLTR, cysteinyl leukotriene receptor; LPAR, lysophosphatidic acid receptor.

Table S2. Classification of class B* GPCRs by helix 8-second residues & subtypes of G proteins.

GPCRs	Helix-8 Second Residue										Predicted Hierarchy or
(signal, G protein subtypes)	all	Glu	Gln	Asp	Asn	His	Lys	Arg	Trp	misc	the 2nd residue, misc.
Glucagon GHRH/GIP/GLP1/GCG Rs*	4	**4**	0	0	0	0	0	0	0	0	VAx(L/I)Y-motif
(hormone, G_s)	100%	**100%**	0%	0%	0%	0%	0%	0%	0%	0%	
Glucagon GLP2 R*	1	**1**	0	0	0	0	0	0	0	0	
(hormone, ?)	100%	**100%**	0%	0%	0%	0%	0%	0%	0%	0%	
Secretin SCT R*	1	**1**	0	0	0	0	0	0	0	0	
(hormone, ?)	100%	**100%**	0%	0%	0%	0%	0%	0%	0%	0%	
Corticotropin releasing hormone $CRF_{1/2}$ Rs*	2	**2**	0	0	0	0	0	0	0	0	VSVFY-motif
(hormone, $G_s > G_{q/11}$)	100%	**100%**	0%	0%	0%	0%	0%	0%	0%	0%	
Calcitonin CT R*	1	**1**	0	0	0	0	0	0	0	0	VAxIY-motif
(peptide, G_s)	100%	**100%**	0%	0%	0%	0%	0%	0%	0%	0%	
Caocitonin receptor-like CALRL R*	1	**1**	0	0	0	0	0	0	0	0	VSxIY-motif
(peptide, –)	100%	**100%**	0%	0%	0%	0%	0%	0%	0%	0%	
PAC1 R*	1	**1**	0	0	0	0	0	0	0	0	Vax(L/I)Y-motif
(polypeptide, G_s)	100%	**100%**	0%	0%	0%	0%	0%	0%	0%	0%	
VIP1/2 Rs*	2	**2**	0	0	0	0	0	0	0	0	
(polypeptide, G_s)	100%	**100%**	0%	0%	0%	0%	0%	0%	0%	0%	
Parathyroid hormone R1*	1	**1**	0	0	0	0	0	0	0	0	
(peptide, $G_s > G_{q/11}$)	100%	**100%**	0%	0%	0%	0%	0%	0%	0%	0%	
Parathyroid hormone R2*	1	**1**	0	0	0	0	0	0	0	0	VSxIY-motif
(peptide, G_s, $G_{q/11}$)	100%	**100%**	0%	0%	0%	0%	0%	0%	0%	0%	
total number of Rs	15	total number of subgroups									10
R rate in the largest subgroups: 15/15	100%	rate of conserved subgroups for the 2nd aa of helix 8: 10/10									100%

GHRHR*, growth hormone releasing hormone receptor; GIPR*, gastric inhibitory polypeptide receptor; GLP1/2R*, glucagon-like peptide-1/2 receptor; GCGR*, glucagon receptor; CT*, Calcitonin receptor; CALRL*, calcitonin receptor-like; SCTR*, secretin receptor; PAC1, pituitary adenylate cyclase activating polypeptide1; CRF, corticotropin-releasing factor; VIPR1/2*, vasoactive intestinal polypeptide receptor1/2; PTH1/2*, parathynoid hormone receptor1/2.

Human GPCRs		Human GPCRs		Subclass.G-pr_subtypes
mOR-S6	*TM7-NPxxY helix 8*	*mOR-S6*	*TM7-NPxxY helix 8*	
GHRHR*(382)	VAILYCFLNQ**E**VRTEISRKWH	GIPR*(398)	VSVLYCFINK**E**VQSEIRRGWH	GlucR.G_s
GLP1R*(408)	VAVIYCFVNN**E**VQLEFRKSWE	GCGR*(406)	VAVLYCFLNK**E**VQSELRRRWH	GlucR.G_s
GLP2R*(442)	VAILYCFLNQ**E**VRTEISRKWH			Glucagon-like peptide 2 R.−
CT*(397)	VATIYCFCNN**E**VQTTVKRQWA			Calcitonin R.$G_s>G_q$
CALRL*(390)	VSTIFCFFNG**E**VQAILRRNWN			Calcitonin gene-related peptide type1 R.−
SCTR*(394)	VAVLYCFLNG**E**VQLEVQKKWQ			Secretin R.−
CRF1*(398)	VSVFYCFLNS**E**VRSAIRKRWH	CRF2*(365)	VSVFYCFFNG**E**VRSAVRKRWH	CRF R.$G_s>G_q$
PAC1*(406)	VAVLYCFLNG**E**VQAEIKRKWR			PAC1 R.G_s
VIP1*(394)	VAILYCFLNG**E**VQAELRRKWR	VIP2*(381)	VAVLYCFLNS**E**VQCELKRKWR	VIP R.G_s
PTH1*(465)	VAIIYCFCNG**E**VQAEIKKSWS			Parathynoid hormone R.$G_s>G_q$
PTH2*(419)	VSIIYCYCNG**E**VQAEVKKMWS			Parathynoid hormone R.G_s/G_q

Supplementary Fig. S2. Alignment of amino acid sequences of NPxxY motif and helix 8 of class B* GPCRs. The 15 human GPCRs are shown. The **bold residue** indicates the 2nd one of helix 8, the position of which in the amino acid sequence is shown in the parentheses. Helix 8 was expected to be formed by hydrophobic residues at the 3rd and more than two of the 7th, 8th, 10th, and 11th positions. The conserved TM7 motif is V(A/S)xxY instead of NPxxY. GHRHR*, growth hormone releasing hormone receptor; GIPR*, gastric inhibitory polypeptide receptor; GLP1/2R*, glucagon-like peptide-1/2 receptor; GCGR*, glucagon receptor; CT*, Calcitonin receptor; CALRL*, calcitonin receptor-like; SCTR*, secretin receptor; PAC1*, pituitary adenylate cyclase activating polypeptide1; CRF, corticotropin-releasing factor; VIPR1/2*, vasoactive intestinal polypeptide receptor1/2; PTH1/2*, parathynoid hormone receptor1/2.

Table S3. Classification of class C** GPCRs by helix 8-second residues & subtypes of G proteins.

GPCRs	Helix-8 Second Residue										Predicted Hierarchy or
(signal, G protein subtypes)	all	Glu	Gln	Asp	Asn	His	Lys	Arg	Trp	misc	the 2nd residue, misc.
Calcium CAS R**	1	0	0	0	**1**	0	0	0	0	0	IYIIL-motif, short helix 8 with weak H-core
(ion, $G_{i/o}$, $G_{q/11}$, $G_{12/13}$)	100%	0%	0%	0%	**100%**	0%	0%	0%	0%	0%	
Calcium CAS(GPR6a) R**	1	**1**	0	0	0	0	0	0	0	0	PKCY(M/V/L)-motif
(ion, $G_{q/11}$)	100%	**100%**	0%	0%	0%	0%	0%	0%	0%	0%	
GABA GABA$_B$1/2 Rs**	2	1	0	1	0	0	0	0	0	0	R1(E),R2(D), PK(C/L)(Y/I)(V/T)-motif?
(neurotransmitter, –)	100%	50%	0%	50%	0%	0%	0%	0%	0%	0%	
Metabotropic glutamate mGlu$_{1/5}$ Rs**	2	0	0	0	**2**	0	0	0	0	0	(V/M)YII(I/L)-motif
(amino acid, $G_{q/11}$ > G_s)	100%	0%	0%	0%	**100%**	0%	0%	0%	0%	0%	
Metabotropic glutamate mGlu$_{2/3/4/6/7/8}$ Rs**	6	0	0	0	**6**	0	0	0	0	0	(V/L/T)(Y/H)(I/V)I(L/I)-motif
(amino acid, $G_{i/o}$)	100%	0%	0%	0%	**100%**	0%	0%	0%	0%	0%	
Umami tastant TAS1R1 R**	1	0	0	**1**	0	0	0	0	0	0	PKCY(M/V/L)-motif
(tastant, –)	100%	0%	0%	**100%**	0%	0%	0%	0%	0%	0%	
Sweet tastant TAS1R2 R**	1	**1**	0	0	0	0	0	0	0	0	PKCY(M/V/L)-motif
(tastant, –)	100%	**100%**	0%	0%	0%	0%	0%	0%	0%	0%	
Tastant-common TAS1R3 R**	1	0	0	0	0	0	0	0	0	**1**	R3(G), PKCY(M/V/L)-motif
(tastant, –)	100%	0%	0%	0%	0%	0%	0%	0%	0%	**100%**	
total number of Rs	15	total number of subgroups									7
R rate in the largest subgroups: 14/15	93%	rate of conserved subgroups for the 2nd aa of helix 8: 6/7									86%

CAS, calcium-sensing; GABA, γ-amino butyric acid, mGlu, metabotropic glutamate; TAS1, taste 1.

Human GPCRs		Human GPCRs		Subclass.G-pr_subtypes
mOR-S6	*TM7-NPxxY* *helix 8*	*mOR-S6*	*TM7-NPxxY* *helix 8*	
CASR**(867)	IYIILFKPSR**N**TIEEVRCS			Calcium Sensing R.G_i,G_q,G_{12}
GPC6a**(842)	PKCYVIICKQ**E**INTKSAFLKM			Calcium Sensing R.G_q
$GABA_{B1}$**(865)	PKMRRLITRG**E**WQSEAQDTMK	$GABA_{B2}$**(752)	PKLITLRTNP**D**AATQNRRFQF	mGluR.G_q
$mGlu_1$**(845)	MYIIIAKPER**N**VRSAFTTSDV	$mGlu_5$**(832)	VYIILAKPER**N**VRSAFTTSTV	mGluR.G_q
$mGlu_2$**(824)	LHIILFQPQK**N**VVSHRAPTSR	$mGlu_3$**(833)	VHIILFQPQK**N**VVTHRLHLNR	mGluR.G_i
$mGlu_4$**(852)	VYIILFHPEQ**N**VPKRKRSLKA	$mGlu_8$**(848)	VYIIIFHPEQ**N**VQKRKRSFKA	mGluR.G_i
$mGlu_6$**(850)	TYVILFHPEQ**N**VQKRKRSLKA	$mGlu_7$**(855)	VYIIIFHPEL**N**VQKRKRSFKA	mGluR.G_i
TAS1R1(820)	PKCYVILCRP**D**LNSTEHFQAS	TAS1R2(820)	PKCYMILFYP**E**RNTPAYFNSM	Taste.−
TAS1R3(822)	PKCYLLMRQP**G**LNTPEFFLGG			Taste.−

Supplementary Fig. S3. Alignment of amino acid sequences of NPxxY motif and helix 8 of class C** GPCRs. The 15 human GPCRs are shown. The **bold residue** indicates the 2nd one of helix 8, the position of which in the amino acid sequence is shown in the parentheses. Helix 8 was expected to be formed by hydrophobic residues at the 3rd and more than two of the 7th, 8th, 10th, and 11th positions.Unstable helix 8 or hydrophobic core. The conserved TM7 motif is PKCYxY, VYIIxF or IYIILF instead of NPxxY. CAS, calcium-sensing; GABA, γ-amino butyric acid, mGlu, metabotropic glutamate receptor; TAS1, taste1.

Table S4. Classification of adhesion class GPCRs by helix 8-second residues & subtypes of G proteins.

GPCRs	Helix-8 Second Residue									Predicted Hierarchy or the 2nd residue, misc.
(signal, G protein subtypes)	all	Glu	Gln	Asp	Asn	His	Lys	Arg	Trp	misc	
ADGRB1/2/3 Rs	3	**3**	0	0	0	0	0	0	0	0	FVIxxxHC-motif
(phosphatidylserine/secretin family?, ?)	100%	**100%**	0%	0%	0%	0%	0%	0%	0%	0%	
ADGRA1/2/3 Rs	3	0	0	**3**	0	0	0	0	0	0	(L/A)F(F/V)xxHHC-motif
(glycosaminoglycans/?, ?)	100%	0%	0%	**100%**	0%	0%	0%	0%	0%	0%	
Cadherin EGF LAG G-type CEL1/2/3 Rs	3	2	0	1	0	0	0	0	0	0	R1/2(E), R3(D), PFxxLx(H/F)C-motif
(cadherin, ?)	100%	67%	0%	33%	0%	0%	0%	0%	0%	0%	
ADGRD1/2 Rs	2	**2**	0	0	0	0	0	0	0	0	L(F/Y)IFL(F/V)(H/Y)(C/A)-motif
(?, G_s/?)	100%	**100%**	0%	0%	0%	0%	0%	0%	0%	0%	
ADGRE R1	1	0	**1**	0	0	0	0	0	0	0	xFIFLxHC-motif
(?, $G_{q/11}$)	100%	0%	**100%**	0%	0%	0%	0%	0%	0%	0%	
ADGRE2/3/P4 Rs	3	0	**3**	0	0	0	0	0	0	0	xFIFLVYC, xLLFVVHC-motif
(?, ?)	100%	0%	**100%**	0%	0%	0%	0%	0%	0%	0%	
ADGRE R5	1	0	0	0	0	0	**1**	0	0	0	xFLYLLHC-motif
(?, $G_{12/13}$)	100%	0%	0%	0%	0%	0%	**100%**	0%	0%	0%	
ADGRF1/3/4/5 Rs	4	0	0	0	0	0	**4**	0	0	0	xFILxFG(C/T)-motif
(?, ?)	100%	0%	0%	0%	0%	0%	**100%**	0%	0%	0%	
ADGRF R2	1	0	0	0	0	0	0	0	0	0	no NPxxY, no helix 8
(?, ?)	100%	0%	0%	0%	0%	0%	0%	0%	0%	0%	
ADGRG R1	1	0	**1**	0	0	0	0	0	0	0	FLIFIWYY-motif
(collagen III, $G_{q/11}$, $G_{12/13}$)	100%	0%	**100%**	0%	0%	0%	0%	0%	0%	0%	
ADGRG R2	1	0	0	0	**1**	0	0	0	0	0	FFILLFYC-motif
(?, $G_{q/11}$)	100%	0%	0%	0%	**100%**	0%	0%	0%	0%	0%	
ADGRG R3	1	0	0	0	0	0	0	0	0	**1**	R3(T), WFxILYLP-motif
(beclometasone dipropionate/?, $G_{i/o}$)	100%	0%	0%	0%	0%	0%	0%	0%	0%	**100%**	
ADGRG 4–7 Rs	4	1	0	0	1	0	0	0	0	2	R7(E), R6(N), G4(S), G5(C), x(F/L)xxx(YLP/FHC)
(?, ?)	100%	25%	0%	0%	25%	0%	0%	0%	0%	50%	
ADGRL R1	1	0	0	0	0	0	**1**	0	0	0	xFIFxFHC-motif
(latrotoxin/?, $G_{q/11}$)	100%	0%	0%	0%	0%	0%	**100%**	0%	0%	0%	
ADGRL 2–4 Rs	3	0	0	0	0	0	**3**	0	0	0	xFIFxFHC-motif
(latrotoxin/FLRT3/?, ?)	100%	0%	0%	0%	0%	0%	**100%**	0%	0%	0%	
ADGRV R1	1	0	0	0	0	0	0	0	0	**1**	R(P), >6000 long sequence
(?, ?)	100%	0%	0%	0%	0%	0%	0%	0%	0%	**100%**	
total number of Rs	33	total number of subgroups									16
R rate in the largest subgroups: 28/33	85%	rate of conserved subgroups for the 2nd aa of helix 8: 13/16									81%

Subtypes of target G proteins were obtained from http://www.guidetopharmacology.org. ADGRA/D/E/F/G/L/V, adhesion G protein-coupled receptor A/D/E/F/G/L/V; ADGRB1/2/3, adhesion G protein-coupled receptor brain-specific angiogenesis inhibitor 1/2/3; CELR, cadherin EGF LAG seven-pass G-type receptors.

Human GPCRs	Human GPCRs	Subclass.G-pr_subtypes
mOR-S6 TM7-NPxxY helix 8	*mOR-S6 TM7-NPxxY helix 8*	
ADGRB1(1190)FVIVMVHCILRR**E**VQDAVKCRVV	ADGRB2(1208)FVITAVHCFLRR**E**VQDVVKCQMG	BraAR.G_i?
ADGRB3(1163)FVIVMVHCILRR**E**VQDAFRCRLR		Brain-specific angiogenesis inhibitor.G_i?
ADGRA1(312) LFVLIHHCAKRE**D**VWQCWWACCP	ADGRA2(1075)LFVFTHHCARRR**D**VRASWRACCP	AdGRA.??
ADGRA3(1062)AFFVVHHCVNRE**D**VRLAWIMTCC		Adhesion GPCR A R.??
CELR1(2707) PFVLLFHCVLNQ**E**VRKHLKGVLG	CELR2(2615) PFIFLSYVVLSK**E**VRKALKLACS	CELR.??
CELR3(2777) LAVLLLFCVLNA**D**ARAAWMPACL		Cadherin EGF LAG seven-pass G-type R.??
ADGRD1(812) LFIFLFHCLLNS**E**VRAAFKHKTK	ADGRD2(907) LYIFLVYAACNE**E**VRSALQRMAE	AdGRD.G_s
ADGRE1(850) AFIFLIHCLLNG**Q**VREEYKWITG		Adhesion GPCR E.G_q
ADGRE2(850) VFIFLVYCLLSQ**Q**VREQYGKWSK	ADGRE3(604) FFIFLVYCILSQ**Q**VQKQYQKWFR	AdGRE.G_q/?
ADGRE4P(441)VLLFVVHCILNR**Q**VRLIILSVIS		Putative Adhesion GPCR P.??
ADGRE5(792) AFLYLLHCLLNK**K**VREEYRKWAC		Adhesion GPCR E R.G_{12}
ADGRF1(844) FFILCFGILLDS**K**LQLLFNSKSA	ADGRF2 no NPxxY, no helix 8	AdGRF.??
ADGRF3(1026)VFILLFGCLMDR**K**IQEALRKRFC	ADGRF4(658) FFILLFGTIMDH**K**IRDALRMRMS	AdGRF.??
ADGRF5(1272)LFILLFGCLWDL**K**VQEALLNKFS		Adhesion GPCR F.??
ADGRG1(664) FLIFIWYWSMRL**Q**ARGGPSPLKS		Adhesion GPCR G.G_q/G_{12}
ADGRG2(885) FFIFIFYCVAKE**N**VRKQWRRYLC		Adhesion GPCR G.G_q
ADGRG3(532) WFTILYLPSQST**T**VSSSTARLDQ		Adhesion GPCR G.G_i
ADGRG4(2992)WFTILYLPSQST**S**VREQWQIHLC	ADGRG7(730) ILYTVRTKVFQS**E**ASKVLMLLSS	AdGRG.??
ADGRG5(508) GFFLFLWFCSQR**C**RSEAEAKAQI	ADGRG6(1170)LFIFIFHCAMKE**N**VQKQWRQHLC	AdGRG.??
ADGRL1(1113)VFIFVFHCALQK**K**VHKEYSKCLR		Adhesion GPCR L.G_q
ADGRL2(1090)VFIFIFHCALQK**K**VRKEYGKCFR		Adhesion GPCR L.??
ADGRL3(1107)MFIFIFHCVLQK**K**VRKEYGKCLR	ADGRL4(670) MFIFLFLCVLSR**K**IQEEYYRLFK	AdGRL.??
ADGRV1(6162)MVYFILHNQMCC**P**MKASYTVEMN		Adhesion GPCR V.??

Supplementary Fig. S4. Alignment of amino acid sequences of NPxxY motif and helix 8 of adhesion class GPCRs. The 33 human GPCRs are shown. The **bold residue** indicates the 2nd one of helix 8, the position of which in the amino acid sequence is shown in the parentheses. Helix 8 was expected to be formed by hydrophobic residues at the 3rd and more than two of the 7th, 8th, 10th, and 11th positions. The conserved TM7 motif is Fx(V/I)xxx(H/Y)C, xFIFxF(H/Y)C or LFIFLx(H/Y)C instead of NPxxY. Unstable helix 8 or hydrophobic core. ADGRA/D/E/F/G/L/V, adhesion G protein-coupled receptor A/D/E/F/G/L/V; ADGRB1/2/3, adhesion G protein-coupled receptor brain-specific angiogenesis inhibitor 1/2/3; CELR, cadherin EGF LAG seven-pass G-type receptors.

Table S5. Classification of Frizzled# GPCRs by helix 8-second residues & subtypes of G proteins.

GPCRs	helix-8 second residue										
(signal, G protein subtypes)	all	Glu	Gln	Asp	Asn	His	Lys	Arg	Trp	misc	the 2nd residue, NPxxY-motif.
FZD1/6 Rs# (Wnt, $G_{i/o}$, $G_{q/11}$)	2<br>100%	0<br>0%	0<br>0%	0<br>0%	0<br>0%	0<br>0%	**2**<br>**100%**	0<br>0%	0<br>0%	0<br>0%	I(T/S)xxFW(I/V)-motif
FZD2/9 Rs# (Wnt, $G_{i/o}$)	2<br>100%	0<br>0%	0<br>0%	0<br>0%	0<br>0%	0<br>0%	**2**<br>**100%**	0<br>0%	0<br>0%	0<br>0%	ITSxFW(I/V)-motif
FZD3 R# (Wnt, G_s)	1<br>100%	0<br>0%	0<br>0%	0<br>0%	0<br>0%	0<br>0%	**1**<br>**100%**	0<br>0%	0<br>0%	0<br>0%	IPSxFWV-motif
FZD4/10 Rs# (Wnt, $G_{12/13}$)	2<br>100%	0<br>0%	0<br>0%	0<br>0%	0<br>0%	0<br>0%	**2**<br>**100%**	0<br>0%	0<br>0%	0<br>0%	ITSxMWI-motif
FZD7 R# (Wnt, G_s, $G_{i/o}$)	1<br>100%	0<br>0%	0<br>0%	0<br>0%	0<br>0%	0<br>0%	**1**<br>**100%**	0<br>0%	0<br>0%	0<br>0%	ITTxFWI-motif
FZD5/8 Rs# (Wnt, ?)	2<br>100%	0<br>0%	0<br>0%	0<br>0%	0<br>0%	0<br>0%	**2**<br>**100%**	0<br>0%	0<br>0%	0<br>0%	ITSxVW(I/V)-motif
SMO R# (oxysterol?, G_s)	1<br>100%	0<br>0%	0<br>0%	0<br>0%	0<br>0%	0<br>0%	0<br>0%	0<br>0%	0<br>0%	**1**<br>**100%**	R(A), TGIxMxW-motif
total number of Rs	11	total number of subgroups									7
R rate in the largest subgroups: 11/11	100%	rate of conserved subgroups for the 2nd aa of helix 8: 7/7									100%

SMO, smoothened.

Human GPCRs		Human GPCRs		Subclass.G-pr_subtypes
mOR-S6	*TM7-NPxxY helix 8*	*mOR-S6*	*TM7-NPxxY helix 8*	
FZD1#(625)	ITSGFWIWSG**K**TLNSWRKFYT	FZD6#(498)	ISAVFWVGSK**K**TCTEWAGFFK	Fzd R.G_i/G_q
FZD2#(543)	ITSGFWIWSG**K**TLHSWRKFYT	FZD9#(532)	ITSGVWVWSS**K**TFQTWQSLCY	Fzd R.G_i/?
FZD3#(502)	IPSVFWVGSK**K**TCFEWASFFH			Frizzled R.G_s
FZD4#(499)	ITSGMWIWSA**K**TLHTWQKCSN	FZD10#(526)	ITSGMWIWTS**K**TLQSWQQVCS	Fzd R.G_{12}
FZD7#(552)	ITTGFWIWSG**K**TLQSWRRFYH			Frizzled R.G_s/G_i
FZD5#(525)	ITSGVWIWSG**K**TVESWRRFTS	FZD8#(608)	ITSGVWVWSG**K**TLESWSLCTR	Fzd R.?
SMO#(540)	TGIAMTWWTK**A**TLLIWRRTWC			Smoothened R.G_i/G_{12}

Supplementary Fig. S5. Alignment of amino acid sequences of NPxxY motif and helix 8 of Frizzled# GPCRs. The 11 human GPCRs are shown. The **bold residue** indicates the 2nd one of helix 8, the position of which in the amino acid sequence is shown in the parentheses. Helix 8 was expected to be formed by hydrophobic residues at the 3rd and more than two of the 7th, 8th, 10th, and 11th positions. Unstable helix 8 or hydrophobic core. The conserved TM7 motif is ITSxFW(I/V) or TGIAMxW instead of NPxxY. SMO, smoothened.

GPCRs		GPCRs		Subclass.G-pr_subtypes
mOR-S6	*TM7_NPxxY helix 8*	*mOR-S6*	*TM7_NPxxY helix 8*	
VN1R1(331)	SPFVLIMSDT**H**ISQFCFACRT	VN1R3(291)	SPFVLMSRHP**R**IPRLGSACCG	VN1R.G_i
VN1R2(376)	SPFVLMCRDP**S**RSRLCSICCR	*m*Vmn1r224(280)	ITAIISICFP**T**LGPFVMNHDS	VN1R.G_i
VN1R4(291)	SPFVLMSCDP**S**VYRFCFAWKR	*m*Vmn1r237(–)	SPFLVISS	VN1R.G_i
VN1R5(326)	SPLMLIYADN**Q**IFKTLQMLWF			Vomeronasal 1 R.G_i
GPR107(545)	TLVFFVLTGY**K**FRPASDNPYL	GPR137(296)	LPTTLLVGFF**R**VHRPPQDLST	??.?
GPR143(317)	SLAFYGWTGC**S**LGFQSPRKEI	GPR157(285)	NCIMFVLCTR**A**VRTRLFSLCC	??.?
*m*Vmn1r1	SPLVLISTEQ**R**MINCLKNTQG	*m*Vmn1r4	TPLVQISSDN**R**IINRLKNLQS	VN1R.G_i
*m*Vmn1r5	TPLVQFSSDN**R**IIIMLKNLQS	*m*Vmn1r6	TPLIQVSFDN**R**IIIMLKNLQS	VN1R.G_i
*m*Vmn1r7	TPLVQISSDN**R**IINMLKNIQS	*m*Vmn1r8	TPLVQISSDN**R**IINMLKNIQS	VN1R.G_i
*m*Vmn1r9	TPLVQISSDN**R**IIIMLKNLQS	*m*Vmn1r10	TPLVQISSDN**R**IIIMLKNLQS	VN1R.G_i
*m*Vmn1r11	TPLVQISSDN**R**IINRLKNLQS	*m*Vmn1r12	TPLVQISSDK**R**ITRMVKNLQS	VN1R.G_i
*m*Vmn1r13	TPLLQISSDK**R**VINVMKTLQS	*m*Vmn1r14	TPFVQISSDT**R**VIRVVKNWHS	VN1R.G_i
*m*Vmn1r15	TPLVQISSDK**R**IINVLKNLQS	*m*Vmn1r16	TPLVQISSEN**R**IITMLKNRQS	VN1R.G_i
*m*Vmn1r17	TPLVQISSDN**R**IIVMLKNMHS	*m*Vmn1r18	SPLVQITSDK**R**IISILKNVHS	VN1R.G_i
*m*Vmn1r19	APLVQISSDK**R**IIHILIHILK	*m*Vmn1r20	SPLVQISSDN**R**IIIMVKNMYS	VN1R.G_i
*m*Vmn1r21	TPVVQISSDK**R**IINVLKNLRS	*m*Vmn1r22	TPLVQISSDN**R**IINVLKNLWL	VN1R.G_i
*m*Vmn1r23	TPLVQISSDK**R**IINVLKNLWL	*m*Vmn1r24	TPLVQITSDE**R**IINVLKNLWP	VN1R.G_i
*m*Vmn1r25	TPLVQISSDK**R**VINVLKNLQS	*m*Vmn1r26	TPLVQISSDN**R**ILKCHQAFFK	VN1R.G_i
*m*Vmn1r27	SPLVQIGSDN**R**IIIMVKNMYS	*m*Vmn1r28	TPLVQITSDK**R**IISILKNMHS	VN1R.G_i
*m*Vmn1r29	TPLVQITSDN**R**IIIMLENMQS	*m*Vmn1r30	TPLVQISSDK**R**VINVLKNSQS	VN1R.G_i
*m*Vmn1r31	TPLVQISSDN**R**IIIMLKNMHS	*m*Vmn1r32	TPLIQIISDN**R**MIITLKNMQK	VN1R.G_i
*m*Vmn1r33	TPLIQISSDN**R**INIMIKNMQK	*m*Vmn1r34	TPLVQISSDK**R**IIIQKKCKNY	VN1R.G_i
*m*Vmn1r35	TPLVQISSDK**R**ISMMLKNMQK	*m*Vmn1r36	TPLVQISSEK**R**IIIILKSMQK	VN1R.G_i
*m*Vmn1r37	TPLVQISSDK**R**IIIILKSMQK	*m*Vmn1r38	TPLIQISSDN**R**ILMILKSMQK	VN1R.G_i
*m*Vmn1r39	TPLIQIISDN**R**IITMLKNMQK	*m*Vmn1r40	NPFVFICTEK**H**IIKFWESKCG	VN1R.G_i
*m*Vmn1r41	SPFVLICTEK**R**MIKFWGSMFG	*m*Vmn1r42	TSYSIELFIM**H**IYATVSPFVF	VN1R.G_i
*m*Vmn1r43	TSYSIELFMI**H**IYATVSPFVF	*m*Vmn1r44	SPFVFICTEK**H**IIKFLRSMCG	VN1R.G_i
*m*Vmn1r45	TSYSIHIFVM**H**IYATVSPFVF	*m*Vmn1r46	SPFVFICTEK**H**IIKFFWSLCG	VN1R.G_i
*m*Vmn1r47	SPFVFMSTEK**H**LVNFFRSMCE	*m*Vmn1r48	SPFVFISTEK**H**IVNILRG	VN1R.G_i
*m*Vmn1r49	SPFVFICTEK**H**IIKFWESIFG	*m*Vmn1r50	SPFVFICNDK**Y**MIKFVTSMCG	VN1R.G_i
*m*Vmn1r51	SPFVFMSTEK**H**IVNCLRSV	*m*Vmn1r52	SPLLVLSNEK**R**ITNLLISMYE	VN1R.G_i
*m*Vmn1r53	SPFVFICTEK**R**ITNFLRSMCG	*m*Vmn1r54	SPFLILSTEK**Y**IINIFRSTFG	VN1R.G_i
*m*Vmn1r55	SPLLLIFRDP**R**GPCSLYFNVG	*m*Vmn1r56	SPLLLIFRDP**R**GHCSLLFSVG	VN1R.G_i
*m*Vmn1r57	SPLLLIFREP**R**GHCSLLFSVG	*m*Vmn1r58	SPFLLIFRDR**K**GHCSLHIIVS	VN1R.G_i
*m*Vmn1r59	SPLLLIFRDP**R**GHCSLLFSVG	*m*Vmn1r60	SPLLLIFRDC**K**GHCSVHIMSV	VN1R.G_i
*m*Vmn1r61	SPLLLIFRDC**K**GHCSVHIMSV	*m*Vmn1r62	SPLLLIFRDS**K**GHCSLHIMSV	VN1R.G_i
*m*Vmn1r63	SPLLLIFRDC**K**GHCSLRIMSV	*m*Vmn1r64	SPFLLICRDP**M**GPCSLLFIVG	VN1R.G_i
*m*Vmn1r65	SPLLLIFRDP**M**CPCPVPFIVG	*m*Vmn1r66	FGPFVLINN-Y**S**VRPRLSLVWM	VN1R.G_i
*m*Vmn1r67	FGPFVLMNHCT**F**VPRLSLIWMW	*m*Vmn1r68	FAPCVLMSHYS**F**MPRFSLVWTW	VN1R.G_i
*m*Vmn1r69	FAPFVLMSHYS**F**MPKLSLTWIR	*m*Vmn1r71	FAPFVLMSHYS**I**VSRLSLVWLR	VN1R.G_i
*m*Vmn1r77	VCPFVLITNM**K**FNFSLFLPCF	*m*Vmn1r80	CPFVLISNMK**P**ISNLFLPCFH	VN1R.G_i
*m*Vmn1r90	SPLLLIFRDP**S**YPCSLIFNYR	*m*Vmn1r168	SPLLLIFRDP**S**YPCSLIFNYK	VN1R.G_i
*m*Vmn1r172	SPLMLIFRGPK**K**ISAKGINTEN	*m*Vmn1r173	SPLMLIFRGPK**K**ISAKGINTEN	VN1R.G_i
*m*Vmn1r174	SPLMLIVRGPK**K**ISAKGINTEN	*m*Vmn1r177	SPLLLIFRDP**S**YHCSLIFNYK	VN1R.G_i
*m*Vmn1r178	SPLLLIFRDP**K**YPCSVLFNC	*m*Vmn1r181	SPLLLIFRDP**K**GPCSVFFNC	VN1R.G_i
*m*Vmn1r184	FGPCVFIKS-Y**S**LMSRCNLAHL	*m*Vmn1r185	FGPCVFMRS-Y**S**LMSRFNLAHL	VN1R.G_i
*m*Vmn1r186	SPLLLIFRDS**K**GHCSLHIMSV	*m*Vmn1r187	SPLLLIFRDC**K**GHCSLRIMSV	VN1R.G_i

Supplementary Fig. S6. Alignment of amino acid sequences of NPxxY motif and helix 8 of vomeronasal 1 and other GPCRs. The 9 human and 113 murine GPCRs are shown. The **bold residue** indicates the 2nd one of helix 8, the position of which in the amino acid sequence is shown in the parentheses. Helix 8 was expected to be formed by hydrophobic residues at the 3rd and more than two of the 7th, 8th, 10th, and 11th positions. The conserved TM7 motif is SPxxL, TLxxF, LPxxL, SLxxY, NCxxF, or ITxII instead of the NPxxY. Unstable helix 8 or hydrophobic core. VN1R, human vomeronasal 1 receptor; *m*Vmn1r, murine vomeronasal 1 receptor; GPR, human other G protein-coupled receptor.

GPCRs		GPCRs		Subclass.G-pr_subtypes
mOR-S6	*TM7_NPxxY helix 8*	*mOR-S6*	*TM7_NPxxY helix 8*	
*m*Vmn1r188	SPFVLIHRDG**L**LAEQWETLKR	*m*Vmn1r189	SPLVLIHKDG**L**LAECWHAQME	VN1R.G_i
*m*Vmn1r191	SPLVLIHRDG**L**LAGCCSAQ	*m*Vmn1r192	SPYVLISRDF**K**VPNVLHAH	VN1R.G_i
*m*Vmn1r197	SPFMLIHRDE**H**VIKCFHTQ	*m*Vmn1r198	SPYVLISRNV**R**VPNTLHAH	VN1R.G_i
*m*Vmn1r199	SPYVLISRNV**R**VPNTLHAH	*m*Vmn1r200	SPLVLIHRDG**L**LVERWHVQWE	VN1R.G_i
*m*Vmn1r201	SPFVLIHRDG**L**LVDWWHAQME	*m*Vmn1r207	SPLVLIHRDG**L**LVECCHAQCE	VN1R.G_i
*m*Vmn1r208	SPFVLIHRDG**L**LSKFWHAHWE	*m*Vmn1r209	SPLVLIHRDG**L**LVECWHAQWE	VN1R.G_i
*m*Vmn1r210	SPFVLIHRDG**L**LVKFWHAQME	*m*Vmn1r217	SPLVLIHRDG**L**LPACWHAQ	VN1R.G_i
*m*Vmn1r220	SPFVLIQRDG**L**IPVCWHAQ	*m*Vmn1r221	SPLVLIHRDR**I**LVECWYVQME	VN1R.G_i
*m*Vmn1r222	SPFVLIHRDG**L**LTEQWETLKQ	*m*Vmn1r225	ITSIISMCFP**T**LGPFVMSHYS	VN1R.G_i
*m*Vmn1r226	ITAIISMCFP**T**LGPIVISPDF	*m*Vmn1r227	ITAIISMCFP**T**LGPIVIGCDF	VN1R.G_i
*m*Vmn1r228	ITAIIALCFP**T**LGPFVMSHDF	*m*Vmn1r229	ITAIISMGFP**A**IGPFVMSRDF	VN1R.G_i
*m*Vmn1r230	IPFVLMSQSS**P**LSKLCFL	*m*Vmn1r231	ITVIIHLCFP**T**LGPFIVTQDT	VN1R.G_i
*m*Vmn1r232	TTAIISMGFP**T**LGPFVMSRDF	*m*Vmn1r233	CPFLLMSHDS**R**ASSFCLPLKR	VN1R.G_i
*m*Vmn1r335	SPFLLMNHYS**I**ASSHCVPCMR	*m*Vmn1r336	CPFLLMSQDS**R**ISSYLKRNIH	VN1R.G_i
*m*Vmn1rD19	SPLLLTFRDP**K**GPCSVFFNC			Vomeronasal 1 R.G_i

Supplementary Fig. S6. Alignment of amino acid sequences of NPxxY motif and helix 8 of vomeronasal 1 and other GPCRs (continued). The 9 human and 113 murine GPCRs are shown. The **bold residue** indicates the 2nd one of helix 8, the position of which in the amino acid sequence is shown in the parentheses. Helix 8 was expected to be formed by hydrophobic residues at the 3rd and more than two of the 7th, 8th, 10th, and 11th positions. The conserved TM7 motif is SPxxL, TLxxF, LPxxL, SLxxY, NCxxF, or ITxII instead of the NPxxY. Unstable helix 8 or hydrophobic core. VN1R, human vomeronasal 1 receptor; *m*Vmn1r, murine vomeronasal 1 receptor; GPR, human other G protein-coupled receptor.

Human GPCRs	Human GPCRs	Subclass.G-pr_subtypes
mOR-S6 TM7-NPxxY helix 8	*mOR-S6 TM7-NPxxY helix 8*	*agonist*
TA2R1(283) HSLILILGNP**K**LKQNAKKFLL		Taste2 R.G_{t3} Peptide
TA2R3(290) HSFILILGNS**K**LKQTFVVMLR		Taste2 R.G_{t3} Chloroquine
TA2R4(286) HSVLIIITHP**K**LKTTAKKILC		Taste2 R.G_{t3} Colchicine
TA2R5(278) HSLILIMGIP**R**VKQTCQKILW		Taste2 R.G_{t3} 1,10-Phenanthroline
TA2R7(291) HSFILILGNNKLRHASLKVIW		Taste2 R.G_{t3} Papaverine
TA2R8(291) HSLILIVLNN**K**LRQTFVRMLT	TA2R41(289) HPFILIFSNL**K**LRSVFSQLLL	Tas2 R.G_{t3} Chloramphenicol
TA2R9(288) HSFILIMGNS**K**LREAFLKMLR		Taste2 R.G_{t3} Pirenzepine
TA2R10(283) HSFILILGNS**K**LKQASLRVLQ		Taste2 R.G_{t3} Strychrine
TA2R13(287) HSFLLILGNA**K**LRQAFLLVAA	TA2R30(285) HPFILILGNK**K**LKQIFLSVLR	Tas2 R.G_{t3} Denatorium
TA2R14(286) HSCVLILGNK**K**LRQASLSVLL		Taste2 R.G_{t3} Picrotoxinin
TA2R16(282) HSTSLMLSSP**T**LKRILKGKC		Taste2 R.G_{t3} Salicin
TA2R20(285) HSFILIWGNK**T**LKQTFLSVLW		Taste2 R.G_{t3} Cromolyn
TA2R31(285) HPFILIWGNK**K**LKQTFLSVLR	TA2R43(285) HPFILIWGNK**K**LKQTFLSVFW	Tas2 R.G_{t3} Aristolochic acid
TA2R38(303) HAAILISGNA**K**LRRAVMTILL		Taste2 R.G_{t3} PROP/PTC
TA2R39(317) HSILLIQDNP**G**LRRAWKRLQL	TA2R50(285) DSFILIWRTK**K**LKHTFLLILC	Tas2 R.G_{t3} Amarogentin
TA2R40(302) HSVQLILGNP**G**LRRAWKRFQH		Taste2 R.G_{t3} Humulones
TA2R46(285) HPFILIWGNK**K**LKQTFLSVLW		Taste2 R.G_{t3} Absinthin
TA2R19(285) HSFILIMGSR**K**LKQTFLSVLW		Taste2 R.G_{t3} ??
TA2R42(290) HSLILILGNS**K**LRQTAVRLLW		Taste2 R.G_{t3} ??
TA2R45(285) HPFILIWGNK**K**LKQTYLSVLW		Taste2 R.G_{t3} ??
TA2R60(302) HPIILLFSNC**R**LRAVLKSRRS		Taste2 R.G_{t3} ??

Supplementary Fig. S7. Alignment of amino acid sequences of NPxxY motif and helix 8 of taste2 GPCRs. The 25 human GPCRs are shown. The **bold residue** indicates the 2nd one of helix 8, the position of which in the amino acid sequence is shown in the parentheses. Helix 8 was expected to be formed by hydrophobic residues at the 3rd and more than two of the 7th, 8th, 10th, and 11th positions. The conserved TM7 motif is H(S/P)xIL instead of the NPxxY. Unstable helix 8 or hydrophobic core.

Human GPCRs mOR-S6	TM7_NPxxY helix 8	Human GPCRs mOR-S6	TM7_NPxxY helix 8	Subclass.G-pr_subtypes
MAS1(290)	NPFIYFFVGSSKKK**R**FKESLK			Orphan MAS1 proto-oncogene R.G_i/G_q
MRGX1(277)	NPIIYFFVG-**S**FRQRQNRQNL	MRGX3(277)	NPIIYFFVG-**S**FRQRQNRQNL	OclsA.G_q
MRGX4(277)	NPIIYFFVG-**S**FRQRQNRQNL			Orphan Mas-related G protein-coupled R X.G_q
MRGX2(284)	NPIIYFFVG-**S**FRKQWRLQQP			Orphan Mas-related G protein-coupled R X.G_i/G_q
MRGRD(282)	NPVIYFLVGS**R**RSHRLPTRSL			Orphan Mas-related G protein-coupled R.G_i/G_q
MRGRE(275)	KPVVYFCLGS**A**QGRRLPLRLV	MRGRF(297)	KPIVYFLAGR**D**KSQRLWEPLR	OclsA.–
MRGRG(256)	KPLIYSGLGR**Q**PGKREPLRSV			Orphan Mas-related G protein-coupled R.–
MAS1L(324)	NPIIYFFVGSLRKK**R**LKESLR			MAS1 proto-ongogene like R.–
GPR42(282)	DPFVYYFSSS**G**FQADFHELLR			lipid? R.??
GPR4(292)	DPILYCLVNE**G**ARSDVAKALH			Orphan clsA.$G_s/G_i/G_q/G_{12}$
GPR6(345)	NPIIYAFRNQ**E**IQRALWLLLC	GPR12(307)	NPVIYAFRNQ**E**IQKALCLICC	OclsA.G_s/G_i
GPR18(292)	DVILYYIVSK**Q**FQARVISVML	GPR68(292)	DPVLYCFVSE**T**THRDLARLRG	OclsA.G_i/G_q
GPR55(294)	DVFCYYFVIK**E**FRMNIRAHRP			Orphan clsA.G_q/G_{12}
GPR17(331)	DPIMYFFVAE**K**FRHALCNLLC			Orphan clsA.G_i>G_q
GPR3(303)	NPIIYAFRNQ**D**VQKVLWACCC	GPR26(300)	DPFVYSLLRH**Q**YRKSCKEILN	OclsA.G_s
GPR61(347)	NPFFYGCLNR**Q**IRGELSKQFV	GPR65(297)	DPILYCFVTE**T**GRYDMWNILK	OclsA.G_s
GPR78(300)	DPFTYSLLRR**P**FRQVLAGMVH	GPR101(460)	HPYVYGYMHK**T**IKKEIQDMLK	OclsA.G_s
GPR132(314)	DPIIYVLATD**H**SRQEVSRIHK			Orphan clsA.G_s
GPR20(303)	DPIVYCFVTS**G**FQATVRGLFG	GPR22(373)	HPLLYAFTRQ**K**FQKVLKSKMK	OclsA.G_i
GPR31(288)	NPVVYCFSSP**T**FRSSYRRVFH	GPR35(282)	DAICYYYMAK**E**FQEASALAVA	OclsA.G_i
GPR33(310)	SPTLYLFVGE**N**FKKVFKKSIL	GPR34(333)	DPVMYFLMSS**N**IRKIMCQLLF	OclsA.G_i
GPR37(555)	TPVLLFCLCK**P**FSRAFMECCC	ETBR2(422)	TPVLLLCICR**P**LGQAFLDCCC	OclsA.G_i
GPR84(376)	NPVLYAAMNR**Q**FRQAYGSILK	GPR183(314)	DPFIYFFACK**G**YKRKVMRMLK	OclsA.G_i
GPR21(310)	NCVIYSISNS**V**FQRGLKRLSG	GPR27(344)	NPVVCFLFNR**E**LRDCFRAQFP	OclsA.G_q
GPR39(350)	NPLLYTVSSQ**Q**FRRVFVQVLC	GPR75(381)	NPFIYSRNSA**G**LRRKVLWCLQ	OclsA.G_q
GPR139(291)	NFFLYCFISK**R**FRTMAAATLK			Orphan clsA.G_q
GPR15(308)	NPFIYYIFDS**Y**IRRAIVHCLC	GPR19(336)	KPTLYSIYNA**N**FRRGMKETFC	OclsA.–
GPR25(313)	NPLIYLLLDR**S**FRARALDGAC	GPR32(322)	NPFLYVFVGR**D**FQEKFFQSLT	OclsA.–
GPR45(330)	NPIVYCWRIK**K**FREACIELLP	GPR52(323)	NCVIYSLSNS**V**FRLGLRRLSE	OclsA.–
GPR50(300)	NAVIYGLLNE**N**FRREYWTIFH	GPR62(296)	HPFLYGLLQR**P**VRLALGRLSR	OclsA.–
GPR63(377)	NPLIYYWRIK**K**FHDACLDMMP	GPR82(316)	DPIIFLLLDK**T**FKKTLYNLFT	OclsA.–
GPR83(351)	NPFIYCWLNE**N**FRIELKALLS	GPR85(345)	NPFVCIFSNR**E**LRRCFSTTLL	OclsA.–
GPR87(320)	DPIIYFFMCR**S**FSRRLFKKSN	GPR88(342)	NPLLYTWRNE**E**FRRSVRSVLP	OclsA.–
GPR135(393)	NPVIYAIRNP**N**ISMLLGRNRE	GPR141(289)	DLLLFVFGGS**H**WFKQKIIGLW	OclsA.–
GPR142(420)	NFGLYCFVSK**T**FRATVRQVIH	GPR146(302)	TPLLYRYMNQ**S**FPSKLQRLMK	OclsA.–
GPR148(326)	LTYLYLLRYR**Q**LLGMVRGHLP	GPR149(366)	TPVFVL--SK**R**WTHLPCGCII	OclsA.–
GPR150(408)	NPFVYLFFQA**G**DCRLRRQLRK	GPR151(312)	NPLIFLVMSE**E**FREGLKGVWK	OclsA.–
GPR152(301)	SPFLCLMASA**D**LRTLLRSVLS	GPR153(302)	LPV-FLWACD**R**YRADLKAVRE	OclsA.–
GPR160(298)	IATVYWFNCH**K**LNLKDIGLPL	GPR161(330)	HPLIYGLWNK**T**VRKELLGMCF	OclsA.–
GPR162(329)	LPS-FIWSCE**R**YRADVRTVWE	GPR171(291)	DPILYY-HSK**A**FRSKVTETFA	OclsA.–
GPR173(346)	NPIVCFLLNK**D**LKKCLRTHAP	GPR174(298)	DPVIYYFSTN**E**FRRRLSRQDL	OclsA.–
GPR176(326)	NPVLFLTVNK**S**VRKCLIGTLV	GPR182(326)	TTLALIFIPK**F**WKLGAPPREE	OclsA.–
LGR4(807)	NPVLYVFFNP**K**FKEDWKLLKR			Orphan clsA.–
LGR5(826)	NPLLYILFNP**H**FKEDLVSLRK	LGR6(833)	NPLLYLLFNP**H**FRDDLRRLRP	OclsA.–

Supplementary Fig. S8. Alignment of amino acid sequences of NPxxY motif and helix 8 of orphan class A GPCRs. The 75 human GPCRs and target G proteins (from http://www.guidetopharmacology. org) are shown. The **bold residue** indicates the 2nd one of helix 8, the position of which in the amino acid sequence is shown in the parentheses. Helix 8 was expected to be formed by hydrophobic residues at the 3rd and more than two of the 7th, 8th, 10th, and 11th positions. Some of their helical structures are likely to be unstable. The conserved TM7 motif is (N/D)Pxx(Y/F). MAS1, MAS1 proto-oncogene G protein-coupled receptor; MAS1L, MAS1 proto-oncogene like, G protein-coupled receptor: MRGR, MAS-related G protein-coupled receptor; MRGX, MAS-related G protein-coupled receptor member X; ETBR2, endothelin B receptor-like protein 2; LGR, leucine-rich repeat containing G protein-coupled receptor.

Human GPCRs		Human GPCRs		Subclass.G-pr_subtypes
mOR-S6	*TM7_NPxxY* *helix 8*	*mOR-S6*	*TM7_NPxxY* *helix 8*	
GPR156**(311)	TTINCFIFIP**Q**LKQWKAFEEE	GPR158**(666)	TVTIGLLLIP**K**FSHSSNNPRDD	OclsC.–
GPR179**(630)	TTTLALIFIP**K**FWKLGAPPRE			Orphan clsC.–
RAI3**(264)	WVFLLAYVSP**E**FWLLTKQRNP			Orphan retinoic acid-induced protein 3.–
GPC5B**(299)	IPEIHCTLLP**A**LQENTPNYFD			Orphan clsC.–
GPC5C**(342)	KGQSMFVENK**A**FSMDEPVAAK	GPC5D**(288)	NACPVTAYQH**S**FQVENQELS	OclsC.–
GPC6A**(842)	PKCYVIICKQ**E**INTKSAFLKM			Orphan clsC.–

Supplementary Fig. S9. Alignment of amino acid sequences of NPxxY motif and helix 8 of orphan class C** GPCRs. The 8 human GPCRs and target G proteins (from http://www.guidetopharmacology. org) are shown. The **bold residue** indicates the 2nd one of helix 8, the position of which in the amino acid sequence is shown in the parentheses. Helix 8 was expected to be formed by hydrophobic residues at the 3rd and more than two of the 7th, 8th, 10th, and 11th positions. Some of their helical structures are likely to be unstable. The conserved TM7 motif is PKCYxI or TTTxxL instead of the NPxxY. RAI3, retinoic acid-induced protein 3; GPC, G protein-coupled receptor family C member.

Human G proteins

```
G proteins                          α4      loop              S6......     α5    _C-terminal
                                    G.H4.23
                                           G.h4s6.3
                                              G.h4s6.10
                                                G.h4s6.12
                                                         G.h4s6.20
                                                                    G.H5.2
                                                                      G.H5.13
GNAS (343,350,354,356,381)  DEFLR  T  D RH------YCYPHFTCAVDTE DIIQRMHLRQYELL
GNAL (330,337,341,343,368)  DLFLR  T  D KH------YCYPHFTCAVDTE DIIQRMHLKQYELL
GNAI1(305,312,316,318,341)  CQFED  K  T E--------IYTHFTCATDTK DVIIKNNLKDCGLF
GNAI2(306,313,317,319,342)  SKFED  K  T E--------IYTHFTCATDTK DVIIKNNLKDCGLF
GNAI3(305,312,316,318,341)  CQFED  R  T E--------IYTHFTCATDTK DVIIKNNLKECGLY
GNAT1(301,308,312,314,337)  VQFLE  M  V E--------IYTHMTCATDTQ DIIIKENLKDCGLF
GNAT2(305,312,316,318,341)  SQFLD  M  V E--------IYSHMTCATDTQ DIIIKENLKDCGLF
GNAT3(305,312,316,318,341)  NQFLD  L  D E--------IYTHMTCATDTQ DIIIKENLKDCGLF
GNAO (306,313,316,318,341)  AQFES  R  N E--------IYCHMTCATDTN DIIIANNLRGCGLY
GNAZ (306,313,317,319,342)  RQFED  R  T E--------IYSHFTCATDTS DVIIQNNLKYIGLC
GNAQ (311,318,323,325,346)  KMFVD  P  D DK------IIYSHFTCATDTE DTILQLNLKEYNLV
GNA11(311,318,323,325,346)  KMFVD  P  D DK------IIYSHFTCA--TE DTILQLNLKEYNLV
GNA14(307,314,317,319,342)  KLYQD  P  D EK------VIYSHFTCATDTD DTILQLNLREFNLV
GNA15(315,321,328,330,361)  MYTRM  T  E SKKGARSRRLFSHYTCATDTQ DSVLARYLDEINLL
GNA12(335, - ,342,344,368)  CFDRK  -  N S------KPLFHHFTTAIDTE DTILQENLKDIMLQ
GNA13(330, - ,337,339,364)  CFRNK  -  D QQ-----KPLYHHFTTAINTE DTILHDNLKQLMLQ
```

Supplementary Fig. S10. Alignment of amino acid sequences for the signature parts in C-terminal regions of α4 and α5 of 16 human G proteins. For each G protein, G.H4.23–27 (α4), G.h4s6.3 and G.h4s6.10 (loop), G.h4s6.12–G.H5.2 and G.H5.13–26 (α5) are shown (the common Gα numbering (CGN) system) [12]. The positions of the first residue are shown in the parentheses. Some of them are considered as residues for the selectivity barcode or precoupling (G.h4s6.10, G.h4s6.12, G.H5.2, G.H5.21) [3] or initial, transient and specific interaction (G.H5.21) or non-specific loop–helix interaction (G.h4s6.10) [10] or M3R–Gα_q interaction (G.h4s6.12) [13] to GPCRs. The C-terminal 6th and charged residue (red or blue) is predicted for a single residue for the determinant of initial, transient and specific interaction between Gα and GPCRs [10]. In this study, the C-terminal 5th and negatively-charged residue (blue) is predicted for the residue for the initial, transient and specific interaction to helix-8-2nd-Arg/Lys/His GPCRs.

Table S6. Classification of non-olfactory class-A GPCRs by helix 8-second residues and subtypes of G proteins

		Helix-8 second residue									
	all	Glu	Gln	Asp	Asn	His	Lys	Arg	Trp	misc	2nd residue
non-olfactory class A GPCRs	21	**7**	0	**8**	0	0	0	2	0	4	S(2), P(2)
rate (G_s)	100%	**33%**	0%	**38%**	0%	0%	0%	10%	0%	19%	
non-olfactory class A GPCRs	79	9	4	11	**18**	1	**19**	7	0	10	S(6), G(2), P(1), I(1)
rate ($G_{i/o}$)	100%	12%	5%	14%	**23%**	1%	**25%**	9%	0%	10%	
non-olfactory class A GPCRs	36	**7**	0	0	4	**5**	2	**5**	0	**13**	S(5), A(3), T(2), I(1), G(1), V(1)
rate ($G_{q/11}$)	100%	**19%**	0%	0%	11%	**14%**	6%	**14%**	0%	**36%**	
non-olfactory class A GPCRs	2	0	0	0	0	0	0	0	0	2	T(1), A(1)
rate ($G_s > G_{i/o}$)	100%	0%	0%	0%	0%	0%	0%	0%	0%	100%	
non-olfactory class A GPCRs	2	0	0	0	0	0	0	0	0	**2**	T(1), A(1)
rate ($G_s > G_{q/11}$)	100%	0%	0%	0%	0%	0%	0%	0%	0%	**100%**	
non-olfactory class A GPCRs	2	**1**	0	**1**	0	0	0	0	0	0	
rate ($G_{i/o} > G_s$)	100%	**50%**	0%	**50%**	0%	0%	0%	0%	0%	0%	
non-olfactory class A GPCRs	5	0	0	1	**2**	0	0	1	0	1	G(1)
rate ($G_{i/o} > G_{q/11}$)	100%	0%	0%	20%	**40%**	0%	0%	20%	0%	20%	
non-olfactory class A GPCRs	6	0	0	1	0	0	0	0	0	**5**	T(4), S(1)
rate ($G_{q/11} > G_s$)	100%	0%	0%	17%	0%	0%	0%	0%	0%	**83%**	
non-olfactory class A GPCRs	6	0	0	0	**2**	0	1	1	0	2	T(1), no-h8(1)
rate ($G_{q/11} > G_{i/o}$)	100%	0%	0%	0%	**33%**	0%	17%	17%	0%	33%	
non-olfactory class A GPCRs	1	0	0	0	**1**	0	0	0	0	0	
rate ($G_{q/11} > G_s/G_{i/o}$)	100%	0%	0%	0%	**100%**	0%	0%	0%	0%	0%	
non-olfactory class A GPCRs	1	0	0	0	0	0	**1**	0	0	0	
rate ($G_{q/11} > G_{12/13}$)	100%	0%	0%	0%	0%	0%	**100%**	0%	0%	0%	
non-olfactory class A GPCRs	2	0	0	0	0	0	0	0	0	**2**	P(1), F(1)
rate (G_s, $G_{i/o}$)	100%	0%	0%	0%	0%	0%	0%	0%	0%	**100%**	
non-olfactory class A GPCRs	2	0	0	0	0	0	0	**2**	0	0	
rate (G_s, $G_{q/11}$)	100%	0%	0%	0%	0%	0%	0%	**100%**	0%	0%	
non-olfactory class A GPCRs	12	**2**	**1**	**2**	**2**	**1**	**2**	0	0	**2**	G(1), L(1)
rate ($G_{i/o}$, $G_{q/11}$)	100%	**17%**	**8%**	**17%**	**17%**	**8%**	**17%**	0%	0%	**17%**	
non-olfactory class A GPCRs	2	**1**	0	**1**	0	0	0	0	0	0	
rate ($G_{i/o}$, $G_{12/13}$)	100%	**50%**	0%	**50%**	0%	0%	0%	0%	0%	0%	
non-olfactory class A GPCRs	3	**1**	0	0	0	0	**1**	0	0	**1**	G(1)
rate ($G_{q/11}$, $G_{12/13}$)	100%	**33%**	0%	0%	0%	0%	**33%**	0%	0%	**33%**	
non-olfactory class A GPCRs	1	0	0	0	0	0	0	0	0	**1**	T(1)
rate (G_s, $G_{i/o}$, $G_{12/13}$)	100%	0%	0%	0%	0%	0%	0%	0%	0%	**100%**	
non-olfactory class A GPCRs	1	0	0	**1**	0	0	0	0	0	0	
rate (G_s, $G_{q/11}$, $G_{12/13}$)	100%	0%	0%	**100%**	0%	0%	0%	0%	0%	0%	
non-olfactory class A GPCRs	3	**3**	0	0	0	0	0	0	0	0	
rate ($G_{i/o}$, $G_{q/11}$, $G_{12/13}$)	100%	**100%**	0%	0%	0%	0%	0%	0%	0%	0%	
non-olfactory class A GPCRs	6	0	0	0	1	0	**2**	1	0	**2**	T(2)
rate (G_s, $G_{i/o}$, $G_{q/11}$)	100%	0%	0%	0%	17%	0%	**33%**	17%	0%	**33%**	
non-olfactory class A GPCRs	1	0	0	0	0	0	0	0	0	**1**	S(1)
rate (G_s, $G_{i/o}$, $G_{q/11}$, $G_{12/13}$)	100%	0%	0%	0%	0%	0%	0%	0%	0%	**100%**	
non-olfactory class A GPCRs	194	**31**	5	**26**	**30**	7	**28**	19	0	48	
rate (misc)	100%	**16%**	3%	**13%**	**15%**	4%	**14%**	10%	0%	25%	
class B* GPCRs	7	**7**	0	0	0	0	0	0	0	0	
rate (G_s)	100%	**100%**	0%	0%	0%	0%	0%	0%	0%	0%	
class B* GPCRs	4	**4**	0	0	0	0	0	0	0	0	
rate ($G_s > G_{q/11}$)	100%	**100%**	0%	0%	0%	0%	0%	0%	0%	0%	
class B* GPCRs	1	**1**	0	0	0	0	0	0	0	0	
rate (G_s, $G_{q/11}$)	100%	**100%**	0%	0%	0%	0%	0%	0%	0%	0%	
class B* GPCRs	12	**12**	0	0	0	0	0	0	0	0	
rate (G_s, $G_s > G_{q/11}$, $G_s/G_{q/11}$)	100%	**100%**	0%	0%	0%	0%	0%	0%	0%	0%	
class C** GPCRs	6	0	0	0	**6**	0	0	0	0	0	
rate ($G_{i/o}$)	100%	0%	0%	0%	**100%**	0%	0%	0%	0%	0%	
class C** GPCRs	5	**2**	0	1	**2**	0	0	0	0	0	
rate ($G_{q/11}$)	100%	**40%**	0%	20%	**40%**	0%	0%	0%	0%	0%	
class C** GPCRs	1	0	0	0	**1**	0	0	0	0	0	
rate ($G_{i/o}$, $G_{q/11}$, $G_{12/13}$)	100%	0%	0%	0%	**100%**	0%	0%	0%	0%	0%	
class C** GPCRs	12	2	0	1	**9**	0	0	0	0	0	
rate (misc)	100%	17%	0%	8%	**75%**	0%	0%	0%	0%	0%	
